# Efficient Genome Editing in *Populus* Using CRISPR/Cas12a

**DOI:** 10.3389/fpls.2020.593938

**Published:** 2020-11-19

**Authors:** Yi An, Ya Geng, Junguang Yao, Chunxiang Fu, Mengzhu Lu, Chun Wang, Juan Du

**Affiliations:** ^1^State Key Laboratory of Subtropical Silviculture, School of Forestry and Biotechnology, Zhejiang A&F University, Hangzhou, China; ^2^State Key Laboratory of Plant Physiology and Biochemistry, College of Life Sciences, Zhejiang University, Hangzhou, China; ^3^State Key Laboratory of Rice Biology, China National Rice Research Institute, Chinese Academy of Agricultural Sciences, Hangzhou, China; ^4^Shandong Provincial Key Laboratory of Energy Genetics, Key Laboratory of Biofuels, Qingdao Institute of Bioenergy and Bioprocess Technology, Chinese Academy of Sciences, Qingdao, China

**Keywords:** CRISPR, Cas12a, genome editing, heat stress, *Populus*, *PagPDS*

## Abstract

The ability to create targeted mutations using clustered regularly inter-spaced short palindromic repeats (CRISPR)/CRISPR-associated (Cas) 9 in support of forest tree biotechnology is currently limited. CRISPR/Cas12a is a novel CRISPR effector protein that not only broadens the CRISPR/Cas targeting range but also enables the generation of large-fragment deletions. In this study, a CRISPR/Cas12a system was evaluated for the induction of targeted mutations in the woody tree poplar (*Populus alba* × *Populus glandulosa*). Three Cas12a nucleases, namely, AsCas12a (*Acidaminococcus* sp. BV3L6), LbCas12a (Lachnospiraceae bacterium ND2006), and FnCas12a (*Francisella tularensis* subsp. *novicidain* U112), were used. We knocked out multiple targets of the *phytoene desaturase gene 8* (*PDS*) using the CRISPR/Cas12a genome-targeting system, and the results indicated that the AsCas12a system is the most efficient. We further optimized the co-cultivation temperature after *Agrobacterium*-mediated transformation from 22 to 28°C to increase the Cas12a nuclease editing efficiency in poplar. AsCas12a showed the highest mutation efficiency, at 70%, and the majority of editing sites were composed of large-fragment deletions. By using this simple and high-efficiency CRISPR/Cas12a system, multiple targets can be modified to obtain multigene simultaneous knockout mutants in tree species, which will provide powerful tools with which to facilitate genetic studies of forest trees.

## Introduction

*Populus* species are of economic and ecological value for paper, timber, construction materials, and biofuel production (Jansson and Douglas, 2007). *Populus* species are also excellent model woody plants for studying perennial secondary vascular cambium activity, wood formation, and secondary cell wall deposition. Functional genomics studies and understanding the developmental regulation of wood formation, especially in wood biomass accumulation and wood property control, are largely hampered by such species’ long life spans, perennial secondary growth, and heterozygous nature (Polle et al., 2013). Due to the limited genetic tools available, it is difficult to make mutants and thus understand gene function through genetic approaches, such as to creating a large number of mutants with carrying single or multiple gene disruptions.

Genome editing technologies are powerful tools for the study of plant gene function and improvement. Genome editing facilitates the process of generating mutations in target genes and shows great potential in expediting improvements in plant health and productivity (White et al., 2007; Strauss et al., 2019). The discovery, development, and application of engineered nucleic acid-binding proteins, including zinc-finger nucleases and transcription activator-like effector nucleases, are rich in notable achievements. Transformative discoveries have shaped the clustered regularly inter-spaced short palindromic repeats (CRISPR)/CRISPR-associated (Cas) toolbox, a class 2/type II CRISPR effector for genetic manipulation on the basis of simpler RNA-guided DNA recognition. A synthetic single-guide RNA (sgRNA), composed of a CRISPR RNA (crRNA) and a *trans*-activating small RNA, guides Cas9 to target genes and generates double-stranded DNA breaks, which can lead to gene mutations that result from non-homologous end-joining repair. The CRISPR/Cas9 system is considered to be a third-generation gene-editing tool. In plants, the earliest reported application of CRISPR/Cas9 technology was in rice (Shan et al., 2013), *Arabidopsis*, and *Nicotiana benthamiana* (Feng et al., 2013; Li et al., 2013; Nekrasov et al., 2013). With its efficiency and simplicity, CRISPR/Cas9 has quickly displaced its predecessors as the method of choice for genome editing (Carroll, 2014). The recently identified CRISPR/Cas12a system is a novel gene-editing tool shown to mediate targeted genome modification in mammalian cells and rice (Zetsche et al., 2015; Hur et al., 2016; Kim et al., 2016). Cas12a, a type V CRISPR effector, recognizes a thymidine-rich protospacer-adjacent motif (PAM) and induces cohesive double-stranded breaks at the target site guided by a single crRNA (Zetsche et al., 2015). The system consists of two components, the Cas12a enzyme and the system-specific crRNA. Although the CRISPR/Cas12a system is similar to the CRISPR/Cas9 system, there are numerous differences: (1) Cas12a recognizes *T*-rich (such as 5′-TTTV-3′) PAM sequences; (2) the PAM sequence is located at the 5′-end of the target DNA sequence, upstream of a protospacer sequence (Fonfara et al., 2016); (3) Cas12a is guided by a single crRNA, and thus no *trans*-acting crRNA is needed (Zetsche et al., 2015); (4) Cas12a shows both DNA and RNA endonuclease activities; and (5) Cas12a contains a RuvC-like domain and a novel nuclease domain, which cleaves the target DNA 18–23 bp downstream of the non-target PAM sequence, resulting in a 5 bp sticky end. The resulting small indel mutations may not prevent the recutting by the same Cas12a/crRNA ribonucleoprotein (RNP). Due to these differences, the CRISPR/Cas12a system induces mutations with a higher probability than Cas9. Because Cas12a generates staggered DSBs away from the seed region, it may promote repeated cleavage and extensive end processing, hence promoting the efficiency of CRISPR/Cpf1 homology directed repair (HDR)-mediated mutations (Wolter and Puchta, 2019; Li et al., 2020).

In recent years, CRISPR/Cas9 genome editing systems have been applied in poplar trees (Fan et al., 2015; Zhou et al., 2015; Azeez and Busov, 2020; Wang et al., 2020). However, it is unknown whether Cas12a works in woody tree species. In this study, we used the hybrid poplar (*Populus alba* × *Populus glandulosa*) clone 84 K to evaluate the targeted mutations *via* the CRISPR/Cas12a system. Three Cas12a nucleases, AsCas12a (*Acidaminococcus* sp. BV3L6), LbCas12a (Lachnospiraceae bacterium ND2006), and FnCas12a (*Francisella tularensis* subsp. *novicidain* U112) (Zetsche et al., 2015), were used and ultimately provided a simple and efficient tool capable of simultaneously editing multiple genes in woody plants. We also optimized the tissue-culture temperature during the transformation of Cas12a. These systems provide a versatile toolbox for studying the functions of multiple genes and gene families in woody plants for basic research and applications in plant gene regulation, epigenomics, and genetic improvement.

## Materials and Methods

### Cloning of *PagPDS* Fragments and CRISPR/Cas12a-Mediated Targeted Mutagenesis of *PagPDS*

Wild-type (WT) poplar 84 K genomic DNA was extracted according to the cetyltrimethylammonium bromide (CTAB) method. *Phytoene desaturase gene 8* (*PDS*) gene was cloned from genomic DNA extracted. Primers to amplify *PagPDS* were designed based on the sequence of the homolog from *Populus trichocarpa* (*Potri.014G148700*) with gene-specific primers (*F*: 5′-GTTTGCAGGGCTGTTGTTACAGTT-3′, R: 5′-CATTTAATGGTGCAGGGAGAACTTCAG-3′). The reaction was amplified for 95°C for 5 min, 35 cycles of 95°C for 30 s, 58°C for 30 s, 72°C for 90 s, and 72°C for 10 min. The PCR product was electrophoresed on an ethidium bromide-stained agarose gel (1%). The DNA was extracted using the TIANgel Midi Purification Kit (Tiangen, Beijing, China) and cloned into the pMD18-T simple vector. The *PagPDS* genomic sequence is shown in Supplementary Figure 1. The sgRNA sequence for *PDS* for the clone 84K was designed based on the basis of the allelic variation of *PagPDS*; five sgRNAs were designed to target five conserved sites and the PAM sequence [FnCas12a, PAM 5′-NTTV-3′, or 5′-TTV-3′ (Zhong et al., 2018); AsCas12a and LbCas12a, PAM 5′-TTTV-3′ (*V* = A, C, G) (Tang et al., 2017)].

### Vector Construction

We codon-optimized *AsCas12a*, *LbCas12a*, and *FnCas12a*, which were attached to nuclear localization signals at the 5′ and 3′ ends of the sequence. Five units of tRNA–crRNAs in their mature form, with each unit including one mature direct repeat (DR) (DR of LbCas12a) and a 24 bp guide sequence, were ligated in tandem and driven by the AtU6-26 promoter in the same construct with AsCas12a, LbCas12a, or FnCas12a (Supplementary Sequence 1). The constructed vectors were named *PagPDS-AsCas12a*, *PagPDS-LbCas12a*, and *PagPDS-FnCas12a*, respectively.

### Transformation and Regeneration

The vectors containing CRISPR/Cas12a with the crRNA expression cassettes and the hygromycin phosphotransferase (HPT) gene (selection marker) were transformed into poplar 84 K by *Agrobacterium*-mediated transformation. *Agrobacterium* cells harboring the vectors were harvested *via* centrifugation and resuspended to OD_600_ = 0.3–0.4. Poplar 84K leaf disks were soaked for 20 min on a shaker with the resuspended cells at a room temperature of 22°C. The inoculated leaf disks were co-cultivated at 22°C in the dark for 2–3 days. The leaf disks were washed with double distilled water and cultured on callus induction medium for 10–30 days in the dark (Han et al., 2000; An et al., 2020). The transgenic lines were selected on hygromycin-supplemented medium. Gene-edited plants were vegetatively propagated on half-strength Murashige and Skoog (MS) medium (pH 5.7) containing 0.8% (w/v) agar at room temperature under a 16 h light/8 h dark photoperiod and a light intensity of 50 μmol m^–2^ s^–1^.

### Heat Stress Treatment

*PagPDS-AsCas12a/LbCas12a/FnCas12a* calli were subjected to heat stress treatment (4 cycles of 28°C for 30 h and 22°C for 40 h), then moved after the final cycle, and cultured continuously at 22°C. Morphological and molecular phenotypes were documented after bud induction.

### Detection of Mutations

For the analysis of the mutations in *PagPDS* in transgenic T_0_ plants, genomic DNA was extracted from stable transgenic and WT plants using the CTAB method. The genomic DNA was used as the template for the PCR amplification of the endogenous *PagPDS* fragment with the primers (*F*: AACTGGGTATGCGAAGACTTCC, R: AAGCAAGCACAAGTATGTTGTCAAC). The primers covered the region containing three of the five target sites of the Cas12a system. The PCR products were separated on an ethidium bromide-stained 1% agarose gel. Bands were recovered and cloned into the pMD18-T simple vector. The mutations were identified *via* the Sanger sequencing of the individual clones. All sequence results were compared with the reference sequence of *PagPDS via* alignment in SnapGene.

## Results

### CRISPR/Cas12a Targeted Mutagenesis in *PagPDS*

**FIGURE 1:**
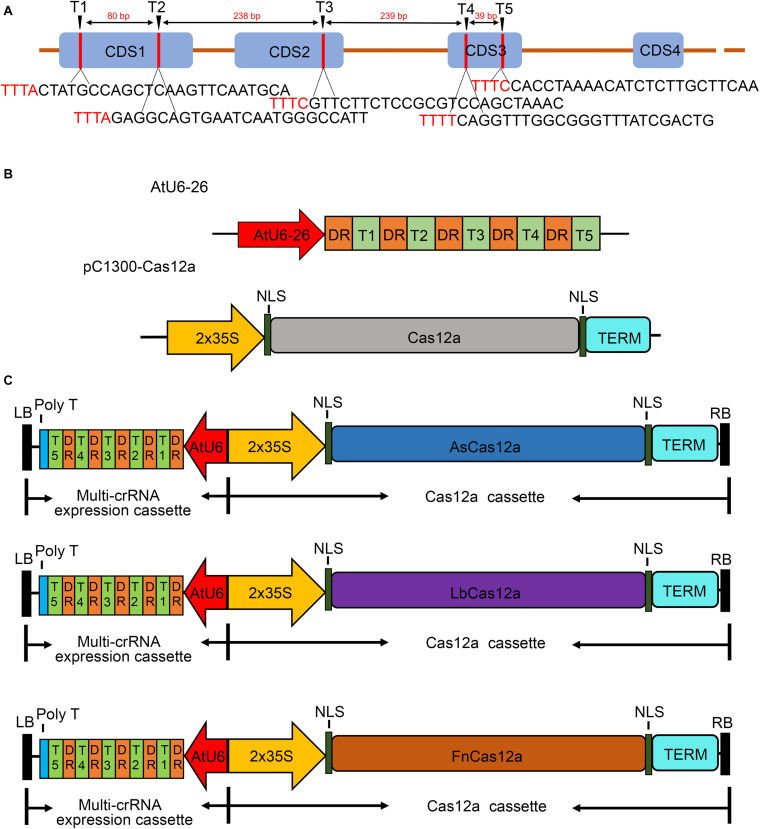
Schematic diagrams illustrating of the PagPDS target sites and the structure of vectors in the CRISPR/Cas12a system. **(A)** Schematic diagrams illustrating the *PagPDS* target sites (T1–T5) and the corresponding. The PAMs (5′-TTTV-3′) are denoted in red. Blue boxes indicate exons (CDS1–CDS4); orange lines indicate introns; the red number indicates the number of nucleotides between different targets. **(B)** Structure of vectors used in the CRISPR/Cas12a system. The intermediate vector AtU6-26 and binary vector pC1300-Cas12a. **(C)** T-DNA region of the constructed gene editing vector.

To test whether the CRISPR/Cas12a system functions in the editing of multiple target sites in poplar 84K, five conserved target sites with the identical target sequences in allelic genes, as well as 5′-TTTV-3′ PAMs located in three exons of *phytoene desaturase gene 8* (*PagPDS*) gene from 84 K, were chosen (Figure 1A). Because Cas12a has the ability to process its own CRISPR RNA (crRNA) and the mature crRNA is sufficient for gene editing, 21 bp mature DRs (DRs of LbCas12a) with five 24 bp guide sequences were ligated in tandem. The multiple crRNA cassettes were driven by *Arabidopsis* RNA polymerase III promoter AtU6-26, whereas *AsCas12a*, *LbCas12a*, and *FnCas12a* were driven by 2 × 35S promoter in pCambia1300 binary vectors (Figure 1B). Three binary vectors, each containing five crRNA expression cassettes, were constructed (Figure 1C).

### Phenotypes of the *PagPDS-AsCas12a/LbCas12a/FnCas12a* Transgenic Plants

**FIGURE 2:**
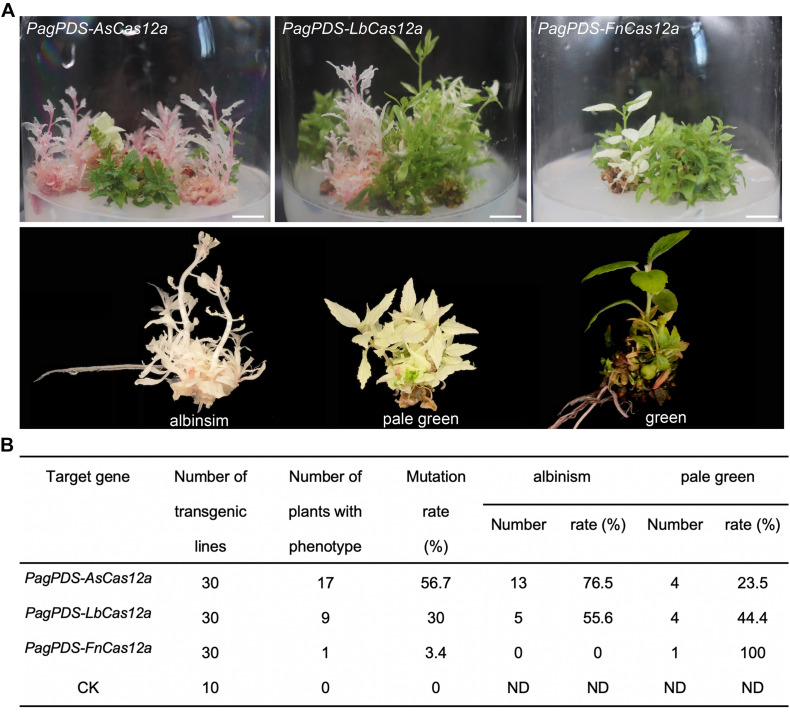
Phenotypes and mutation frequency of the transgenic plants. **(A)** Phenotypes of the *PagPDS-AsCas12a*/*LbCas12a*/*FnCas12a*-mediated transgenic plants. Scale bar = 1 cm. **(B)** Determination of mutation rate in transgenic *T*_0_ poplar plants generated with the CRISPR/Cas12a system under room temperature (22°C). CK, empty vector. ND, not determined.

Thirty transgenic lines were obtained for each *PagPDS-AsCas12a*, *PagPDS-LbCas12a*, and *PagPDS-FnCas12a* constructs. A previous report showed that biallelic homozygous and heterozygous mutants of *PDS* exhibited albino and pale green phenotypes (Fan et al., 2015). In this study, albino and pale green seedlings were observed in all three Cas12a transgenic plants at room temperature (22°C), meaning that mutations were generated at the *PDS* gene and these three Cas12a systems had gene-editing capabilities in poplar (Figure 2A). The proportion of albino seedlings was used to access the efficiency of gene editing, and up to 56.7% of editing was obtained in the AsCas12a system. Among the transgenic plants, 76.5% were albino, and 23.5% were pale green. The efficiency of gene editing was 30% in the LbCas12a system, but only 3.4% in the *PagPDS-FnCas12a* system (Figure 2B). This result was unexpected, as AsCas12a has been reported to show extremely low gene-editing ability in other plants, including rice (Hu et al., 2017; Tang et al., 2017), but exhibited a high editing efficiency in *Populus*.

### Heat Stress Improves CRISPR/Cas12a Efficiency in *Populus*

**FIGURE 3:**
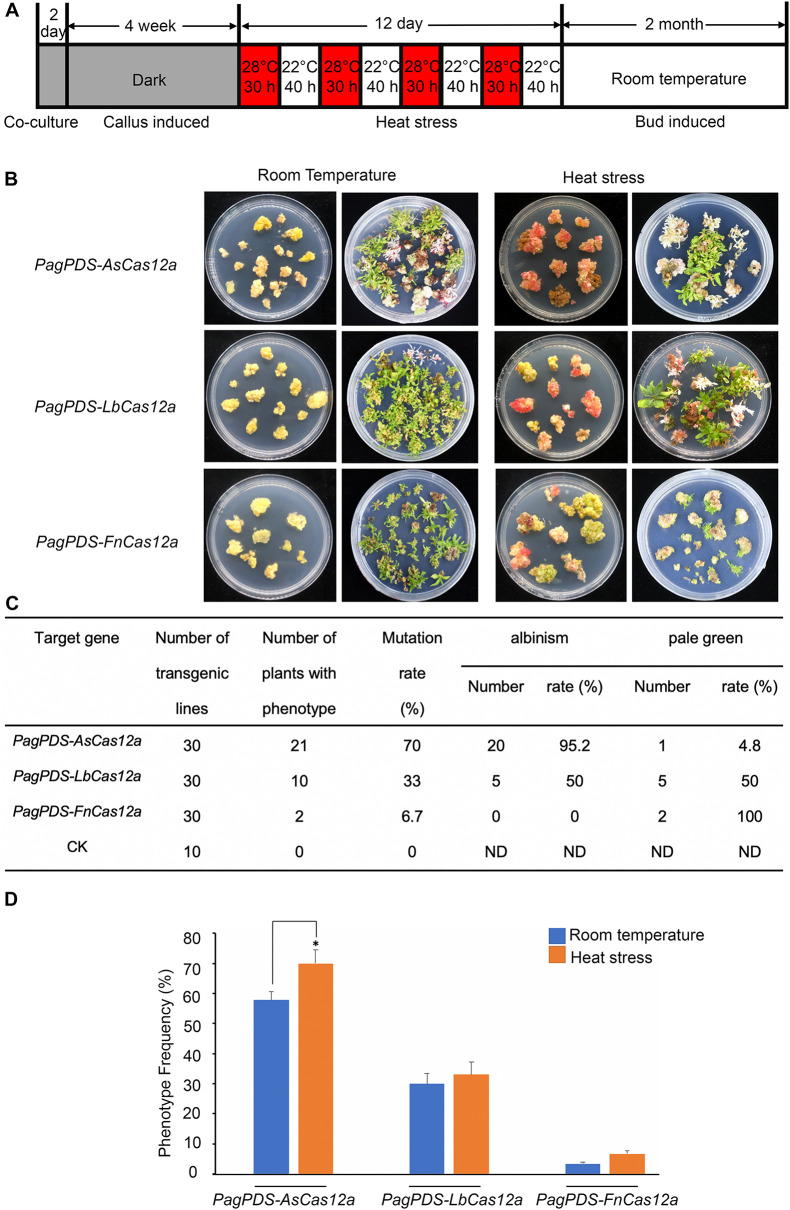
Repeated heat stress treatment increases the efficiency of targeted mutagenesis in calli of 84K poplar. **(A)** Schematic representation of the culture conditions for callus exposure to heat stress at 28°C. **(B)** Phenotypes of the transgenic plants induced with the CRISPR/Cas12a system at room temperature (22°C) and under heat stress (28°C–22°C). Calli were grown for 6 weeks. Bud induced at 1 month. **(C)** Determination of mutation types in transgenic *T*_0_ poplar plants induced with the CRISPR/Cas12a system under heat stress. CK, empty vector. ND, not determined. **(D)** Albinism and pale green plant frequency induced at room temperature (22°C) and under heat stress (28°C–22°C). Data are presented as mean ± SD (*n* = 3 experiments). Each experiment is composed of three biological replicates. Asterisks indicate a significant difference (*P < 0.05; one-way ANOVA; ns, non-significant difference).

To further improve the gene-editing efficiency of Cas12a, we increased the temperature during the transformation process in poplar. Similar to the practice used in *Arabidopsis* (LeBlanc et al., 2018), the calli of 84 K were induced for 4 weeks in the dark from leaf disks soaked in *Agrobacterium* suspension, then subjected to 4 cycles of heat stress treatment (28–22°C), and allowed to recover before shoot induction (Figure 3A). The induced shoots with albino phenotypes were more frequent after heat stress-treated than with no treatment control (Figure 3B). The gene-editing efficiency of the AsCas12a system is still the highest, at up to 70%, 95.2% of which are albino and, 4.8% of which are pale green. The gene-editing efficiency of LbCas12a was about 33%, whereas that of FnCas12a was only 6.7% (Figure 3C). Compared with room temperature (22°C), the phenotype frequency of *PagPDS-AsCas12a* was improved by about 13.3%, that of *PagPDS-LbCas12a* was improved by about 3%, and that of *PagPDS-FnCas12a* was increased by 3.3%, significantly (Figure 3D). Thus, increasing the temperature did improve the editing efficiency of Cas12a.

### *PagPDS* Mutations Match the Designed Target Sites

To evaluate the simultaneous editing of multiple target sites, the genomic DNA of transgenic plants under heat treatment was extracted, and the target sequences were amplified *via* PCR. All mutations generated by Cas12a at the four targets (T1–T4) were deletions, ranging from 1 to 30 nt around sticky overhangs far away the PAM. No mutations were detected at T5.

**FIGURE 4:**
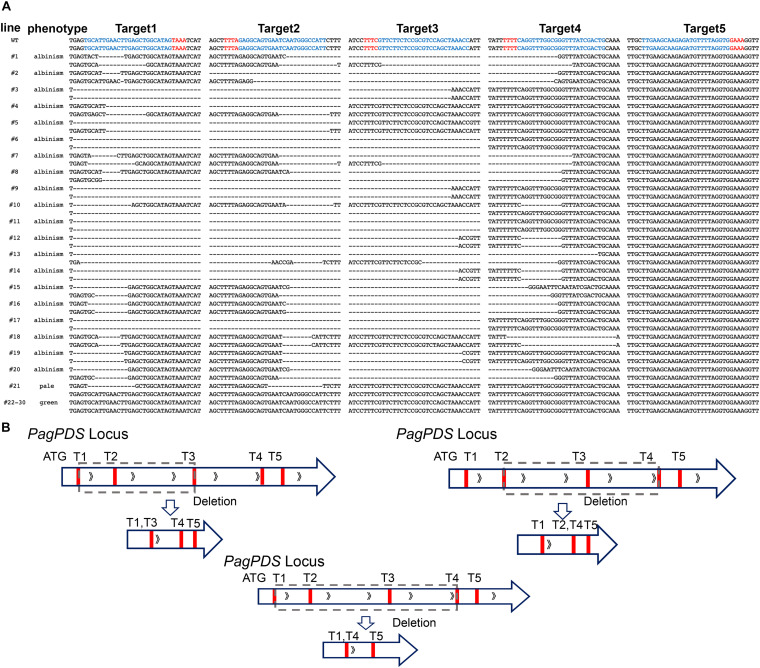
Phenotypes of *T*_0_ transgenic plants of 84K poplar plants with *PagPDS-AsCas12a*. **(A)** All editing events in different target sites in AsCas12a-mediated *T*_0_ plants harvested in this study. Red letters indicate PAM sequences, blue letters indicate target sites, and “–” indicates deletion. **(B)** A large-fragment deletion mutant of *PagPDS*. The sequences from target sites 1 to 3, target sites 1 to 4, and target sites 2 to 4 were lost in the DNA repairing after the Cas12a-mediated break.

In the *PagPDS-AsCas12a-*mediated transgenic lines, gene editing is diverse. Targeting sites and a large-fragment deletion were common in our experiments (Figure 4A). The large-fragment deletion alleles carry mutations at two or four target sites (Figure 4B), and some alleles containing a large deletion between two target sites. The large-fragment deletion frequency reached as high as 60%. Regarding the analyses of T1, mutations in at least one allele were detected in 21 plants (70%), of which 20 had mutations in both alleles, including 9 homozygous mutations and 11 biallelic mutations. At T2, the mutation rate is the same as at T1 (70%), including 10 homozygous mutations and 10 biallelic mutations. The mutation rate is lower at T3 (56.7%), with homozygous mutations in 13 plants and biallelic mutations in 3 plants. At T4, the mutation rate is the lowest (40%), including five homozygous mutations and six biallelic mutations (Table 1). In our CRISPR/Cas12a system, the editing efficiency of the target sites’ approximate 5′-terminus is higher than that of the distant 5′-terminus. No mutations were detected at T5, possibly indicating that the last DR guide unit was incompletely processed during crRNA maturation. Moreover, mutations at multiple sites also occur at the same time. To determine whether the five targets were co-mutated simultaneously or independently, we analyzed the co-mutation rate. Of the *PagPDS-AsCas12a* transgenic lines analyzed, 36.7% carried quadruple-target mutations, 23.3% displayed triple-target mutations, and 10% displayed double-target mutations. In *PagPDS-LbCas12a* transgenic lines, the efficiency of double-target mutations is higher than that of the other targets. In *PagPDS-FnCas12a* transgenic lines, only 6.7% of mutations are double-target mutations. No transgenic line was detected with triple- and quadruple-target mutations (Table 2). The results indicate that AsCas12a has an increased capacity to edit multiple sites simultaneously in the *T*_0_ generation of poplar.

**TABLE 1:** Summary of the mutation types at each target site.

Mutation type	PagPDS-AsCas12a					PagPDS-LbCas12a					PagPDS-FnCas12a				
	T1	T2	T3	T4	T5	T1	T2	T3	T4	T5	T1	T2	T3	T4	T5
Biallelic mutation	11	10	3	6	0	0	3	0	0	0	0	0	0	0	0
Homozygous mutation	9	10	13	5	0	3	2	0	0	0	0	0	0	0	0
Heterozygous mutation	1	1	1	1	0	4	5	4	2	0	2	2	0	0	0
Unmodified	9	9	13	17	30	23	20	26	28	30	28	28	30	30	30
Mutation rate	70%	70%	56.7%	40%	0	23.3%	33.3%	13.3%	6.7%	0	6.7%	6.7%	0	0	0

**TABLE 2:** Examination of the co-mutation frequencies of the five target sites.

		Single	Double	Triple	Quadruple	Quintuple
Heat stress	*PagPDS-AsCas12a*	0	10% (3)	23.3% (7)	36.7% (11)	0
	*PagPDS-LbCas12a*	0	26.7% (8)	3.3% (1)	3.3% (1)	0
	*PagPDS-FnCas12a*	0	6.7% (2)	0	0	0

Although LbCas12a produced a lower editing efficiency than AsCas12a, the edited alleles also included multiple sites and large-fragment deletions. In *PagPDS-FnCas12a*-mediated transgenic plants, only two plants with heterozygous alleles exhibited pale green phenotypes. The gene-editing data are presented in Supplementary Dataset S1.

## Discussion

In this study, we established a CRISPR/Cas12a system to generate stable transgenic poplar trees and optimized the CRISPR–Cas12a system to broaden the genome editing toolbox available for woody plants. Three kinds of codon-optimized Cas12a (Lb, Fn, and As) for rice (Hu et al., 2017) were used in poplar, and these nucleases were driven by 2 × 35 S, with two NLSs at both ends. Because Zetsche et al. (2015) indicate that the DR sequences of the three Cas12a systems are similar and previous study findings show that LbCas12a performed better in the CRISPR/Cas12a system in rice (Tang et al., 2017; Xu et al., 2017; Li et al., 2018), the DR of LbCas12a was chosen as the common DR for all three Cas12a proteins. We tested the robustness of various CRISPR/Cas12a systems in poplar and found that the AsCas12a system with the DR of LbCas12a was the most efficient, even with the DR of Lb. In contrast to the results obtained in rice, AsCas12a has the highest genome editing efficiency in poplar. This result also proves that Cas12a’s ability to recognize the DR is not so specific, and that the AsCas12a and FnCas12a proteins have genome editing capability with the DR of LbCas12a. We believe that Cas12a has different genome editing efficiencies in different species because AsCas12a has a high genome editing efficiency in human cells and mouse cells but with a low efficiency in rice (Zetsche et al., 2015, 2017). *Populus* species are woody plants, which are different from herbs. This may be why the genome editing efficiency of AsCas12a in poplar is high and that in rice is low. The current study has demonstrated high-efficiency multiplex site editing in a woody plant using engineered CRISPR/Cas12a with a simple short DR-guide array. Theoretically, this simple system can be used to edit an unlimited number of genes simultaneously in *Populus*. It is possible to generate specific forms of mutants by means of a multi-target system, for example, a gene mutant that lacks a particular domain or the mutation of an entire gene family. Thus, multiplex site editing provides a powerful tool for targeting members of multigene families.

Nuclease activities are crucial for genome editing efficiency. To improve efficiency, nuclease activities must be optimized by determining their optimal working temperature. Xiang et al. (2017) and LeBlanc et al. (2018) reported a strategy with which to improve Cas9 nuclease activity in *Arabidopsis* by applying heat stress at 37°C. Substantially higher frequencies of CRISPR/Cas9-induced mutations were observed as compared with plants grown continuously at the standard temperature (22°C). The temperature dependency of CRISPR nucleases reflects the fact that they originate from human pathogenic bacteria. Moreno-Mateos et al. (2017) showed that the mutagenic activity of AsCas12a at 28°C in zebrafish embryos increases the severity of the phenotype and the extent of mutant cells. Temperature sensitivity also affects for improved genome editing in rice, maize, and *Arabidopsis* (Lee et al., 2019; Malzahn et al., 2019). However, no reports have described increased Cas12a activity in woody plants grown under comparable conditions. In tissue culture, poplar callus is typically cultured at 22°C. In the present study, we applied the CRISPR/Cas12a system to a woody plant and observed that heating stress at 28°C increased the frequency of gene editing. We showed that temperature modulates Cas12a activity by controlling its ability to access genomic DNA in poplar. At 28°C, the effect is strong on AsCas12a and moderate on LbCas12a, whereas FnCas12a is less affected by temperature. We exploited this property to show that the temporal control of the temperature allows the modulation of Cas12a-mediated genome editing, although the editing efficiency is still relatively low and requires further improvement. We also observed that no new albino-phenotype plants were induced in calli cultured for 2 weeks at 34°C and 37°C. At these temperatures, the calli developed poorly; therefore, we assume that Cas12a functionality may be dependent on the status of the plant cell and the temperature.

The present results demonstrate that Cas12a can be utilized as an alternative system for genome editing in woody plants. Cas12a is a novel CRISPR-effect protein with many different characteristics from Cas9, which may help overcome some of the limitations of CRISPR/Cas9 applications. Cas9 mutants are able to identify atypical PAM sequences, such as 5′-NAG-3′, but the editable range of plant gene targets for the CRISPR/Cas9 system remains limited to guanine-rich loci. Given that the PAM sequence recognized by Cas12a differs–the PAM sequence recognized by FnCas12a is 5′-NTTV-3′ or 5′-TTV-3′ (Zhong et al., 2018), and that recognized by AsCas12a and LbCas12a is 5′-TTTV-3′ (*V* = A, C, and G) (Tang et al., 2017)–the CRISPR/Cas12a system expands the capability to edit plant genomes, especially AT-rich sequences. The longer the PAM sequence, the fewer potential off-target sites in the genome and, hence, the greater the accuracy with which Cas12a identifies the target gene. Compared with the common short indels (1–2 bp) generated by Cas9 in *Populus*, the majority of mutations generated by Cas12a were relatively long, which may be caused by the non-homologous end-joining repair of the 4–5 nt overhangs resulting from the staggered cutting of Cas12a. Given the substantial differences between Cas12a and Cas9 with regard to target recognition and DNA cleavage, CRISPR/Cas12a not only provides a new and alternative method for targeted mutagenesis in plants but also greatly enhances the scope and precision of crop genome editing. There is substantial room for the development, transformation, and utilization of this method.

Because the CRISPR/Cas12a system enables the editing of multiple targets simultaneously in *Populus*, it can be used to knockout the members of a multigene family, which is very important in elucidating gene function due to gene redundancy in woody plants. As an easy and affordable tool, the CRISPR/Cas12a system overcomes the present paucity of mutants of woody plant mutants and shows promise for applications in the fields of gene function and regulation. It may help provide insights into the biological processes unique to woody plants.

## Data Availability Statement

The datasets presented in this study can be found in online repositories. The names of the repository/repositories and accession number(s) can be found in the article/Supplementary Material.

## Author Contributions

JD, CW, and YA design the research experiments. YA performed the experiments and prepared the manuscript. CW constructed the vectors. YG and JY helped in analyzing the sequence data. CF provided technical support. CW, JD, and ML draft the manuscript. All authors contributed to the article and approved the submitted version.

## Conflict of Interest

The authors declare that the research was conducted in the absence of any commercial or financial relationships that could be construed as a potential conflict of interest.

## Supplementary Material

Supplementary Figure 1 *PagPDS* genomic sequences (exon 1–exon 5) from 84K (*Populus alba* × *P. glandulosa*). Blue lines indicate exons. Red lines represent the PAM sequence. Yellow highlights indicate the five target sites. *Potri.014G148700* is *PDS* from *P. trichocarpa*.

Supplementary Dataset 1 LbCas12a- and FnCas12a-mediated gene editing in transgenic poplar plants.

Supplementary Sequence 1 The crRNA expression cassette used in AsCas12a, LbCas12a, and FnCas12a multiplex gene-editing vectors. Letters in red indicate direct repeats (DR). Highlights indicate the five target sites. The letters in blue indicate poly *T*.

## References

[B1] An Y. Zhou Y. Han X. Shen C. Wang S. Liu C. (2020). The GATA transcription factor GNC plays an important role in photosynthesis and growth in poplar. *J. Exp. Bot.* 71 1969–1984. 10.1093/jxb/erz564 31872214 PMC7094078

[B2] Azeez A. Busov V. (2020). CRISPR/Cas9-mediated single and biallelic knockout of poplar STERILE APETALA (PopSAP) leads to complete reproductive sterility. *Plant Biotechnol. J.* 10.1111/pbi.13451 Online ahead of print 32663371 PMC7769228

[B3] Carroll D. (2014). Genome engineering with targetable nucleases. *Annu. Rev. Biochem.* 83 409–439. 10.1146/annurev-biochem-060713-35418 24606144

[B4] Fan D. Liu T. Li C. Jiao B. Li S. Hou Y. (2015). Efficient CRISPR/Cas9-mediated targeted mutagenesis in populus in the first generation. *Sci. Rep.* 5:12217. 10.1038/srep12217 PMC4507398 26193631

[B5] Feng Z. Zhang B. Ding W. Liu X. Yang D. L. Wei P. (2013). Efficient genome editing in plants using a CRISPR/Cas system. *Cell Res.* 23 1229–1232. 10.1038/cr.2013.114 23958582 PMC3790235

[B6] Fonfara I. Richter H. Bratovic M. Le Rhun A. Charpentier E. (2016). The CRISPR-associated DNA-cleaving enzyme Cpf1 also processes precursor CRISPR RNA. *Nature* 532 517–521. 10.1038/nature17945 27096362

[B7] Han K. H. Meilan R. Ma C. Strauss S. H. (2000). An *Agrobacterium tumefaciens* transformation protocol effective on a variety of cottonwood hybrids (genus Populus). *Plant Cell Rep.* 19 315–320. 10.1007/s002990050019 30754915

[B8] Hu X. Wang C. Liu Q. Fu Y. Wang K. (2017). Targeted mutagenesis in rice using CRISPR-Cpf1 system. *J. Genet. Genom.* 44 71–73. 10.1016/j.jgg.2016.12.001 28043782

[B9] Hur J. K. Kim K. Been K. W. Baek G. Ye S. Hur J. W. (2016). Targeted mutagenesis in mice by electroporation of Cpf1 ribonucleoproteins. *Nat. Biotechnol.* 34 807–808. 10.1038/nbt.3596 27272385

[B10] Jansson S. Douglas C. J. (2007). Populus: a model system for plant biology. *Annu. Rev. Plant Biol.* 58 435–458. 10.1146/annurev.arplant.58.032806.103956 17280524

[B11] Kim D. Kim J. Hur J. K. Been K. W. Yoon S.-H. Kim J.-S. (2016). Genome-wide analysis reveals specificities of Cpf1 endonucleases in human cells. *Nat. Biotechnol.* 34 863–868. 10.1038/nbt.3609 27272384

[B12] LeBlanc C. Zhang F. Mendez J. Lozano Y. Chatpar K. Irish V. F. (2018). Increased efficiency of targeted mutagenesis by CRISPR/Cas9 in plants using heat stress. *Plant J.* 93 377–386. 10.1111/tpj.13782 29161464

[B13] Lee K. Zhang Y. Kleinstiver B. P. Guo J. A. Aryee M. J. Miller J. (2019). Activities and specificities of CRISPR/Cas9 and Cas12a nucleases for targeted mutagenesis in maize. *Plant Biotechnol. J.* 17 362–372. 10.1111/pbi.12982 29972722 PMC6320322

[B14] Li J. F. Norville J. E. Aach J. Mccormack M. Zhang D. Bush J. (2013). Multiplex and homologous recombination-mediated genome editing in Arabidopsis and Nicotiana benthamiana using guide RNA and Cas9. *Nat. Biotechnol.* 31 688–691. 10.1038/nbt.2654 23929339 PMC4078740

[B15] Li S. Zhang X. Wang W. Guo X. Wu Z. Du W. (2018). Expanding the Scope of CRISPR/Cpf1-Mediated Genome Editing in Rice. *Mol. Plant* 11 995–998. 10.1016/j.molp.2018.03.009 29567453

[B16] Li S. Zhang Y. Xia L. Qi Y. (2020). CRISPR-Cas12a enables efficient biallelic gene targeting in rice. *Plant Biotechnol. J.* 18 1351–1353. 10.1111/pbi.13295 31730252 PMC7206995

[B17] Malzahn A. A. Tang X. Lee K. Ren Q. Sretenovic S. Zhang Y. (2019). Application of CRISPR- Cas12a temperature sensitivity for improved genome editing in rice, maize, and *Arabriopsis*. *BMC Biol.* 17:9. 10.1186/s12915-019-0629-5 30704461 PMC6357469

[B18] Moreno-Mateos M. A. Fernandez J. P. Rouet R. Vejnar C. E. Lane M. A. Mis E. (2017). CRISPR-Cpf1 mediates efficient homology-directed repair and temperature-controlled genome editing. *Nat. Commun.* 8:2024. 10.1038/s41467-017-01836-2 29222508 PMC5722943

[B19] Nekrasov V. Staskawicz B. Weigel D. Jones J. D. Kamoun S. (2013). Targeted mutagenesis in the model plant Nicotiana benthamiana using Cas9 RNA-guided endonuclease. *Nat. Biotechnol.* 31 691–693. 10.1038/nbt.2655 23929340

[B20] Polle A. Janz D. Teichmann T. Lipka V. (2013). Poplar genetic engineering: promoting desirable wood characteristics and pest resistance. *Appl. Microbiol. Biotechnol.* 97 5669–5679. 10.1007/s00253-013-4940-8 23681587

[B21] Shan Q. Wang Y. Li J. Zhang Y. Chen K. Liang Z. (2013). Targeted genome modification of crop plants using a CRISPR-Cas system. *Nat. Biotechnol.* 31 686–688. 10.1038/nbt.2650 23929338

[B22] Strauss S. H. Boerjan W. Chiang V. Costanza A. Coleman H. Davis J. M. (2019). Certification for gene-edited forests. *Science* 365 767–768. 10.1126/science.aay6165 31439790

[B23] Tang X. Lowder L. G. Zhang T. Malzahn A. A. Zheng X. Voytas D. F. (2017). A CRISPR-Cpf1 system for efficient genome editing and transcriptional repression in plants. *Nat. Plants* 3:17103. 10.1038/nplants.2017.103 28628131

[B24] Wang J. Wu H. Chen Y. Yin T. (2020). Efficient CRISPR/Cas9-mediated gene editing in an interspecific hybrid poplar with a highly heterozygous genome. *Front. Plant Sci.* 11:996. 10.3389/fpls.2020.00996 32719704 PMC7347981

[B25] White T. L. Adams W. T. Neale D. B. (2007). *Forest Genetics.* Wallingford: CABI.

[B26] Wolter F. Puchta H. (2019). In planta gene targeting can be enhanced by the use of CRISPR/Cas12a. *Plant J.* 100 1083–1094. 10.1111/tpj.14488 31381206

[B27] Xiang G. Zhang X. An C. Cheng C. Wang H. (2017). Temperature effect on CRISPR-Cas9 mediated genome editing. *J. Genet. Genom.* 44 199–205. 10.1016/j.jgg.2017.03.004 28412228

[B28] Xu R. Qin R. Li H. Li D. Li L. Wei P. (2017). Generation of targeted mutant rice using a CRISPR-Cpf1 system. *Plant Biotechnol. J.* 15 713–717. 10.1111/pbi.12669 27875019 PMC5425385

[B29] Zetsche B. Gootenberg J. S. Abudayyeh O. O. Slaymaker I. M. Makarova K. S. Essletzbichler P. (2015). Cpf1 is a single RNA-guided endonuclease of a class 2 CRISPR-Cas system. *Cell* 163 759–771. 10.1016/j.cell.2015.09.038 26422227 PMC4638220

[B30] Zetsche B. Heidenreich M. Mohanraju P. Fedorova I. Kneppers J. Degennaro E. M. (2017). Multiplex gene editing by CRISPR-Cpf1 using a single crRNA array. *Nat. Biotechnol.* 35 31–34. 10.1038/nbt.3737 27918548 PMC5225075

[B31] Zhong Z. Zhang Y. You Q. Tang X. Ren Q. Liu S. (2018). Plant Genome Editing Using FnCpf1 and LbCpf1 Nucleases at Redefined and Altered PAM Sites. *Mol. Plant* 11 999–1002. 10.1016/j.molp.2018.03.008 29567452

[B32] Zhou X. Jacobs T. B. Xue L. J. Harding S. A. Tsai C. J. (2015). Exploiting SNPs for biallelic CRISPR mutations in the outcrossing woody perennial Populus reveals 4-coumarate:CoA ligase specificity and redundancy. *New Phytol.* 208 298–301. 10.1111/nph.13470 25970829

