# Supplementary material for: Efficient Genome Editing in Populus Using CRISPR/Cas12a
Source: Front Plant Sci. 2020 Nov 19;11:593938. doi: 10.3389/fpls.2020.593938 (PMC7720674; doi:10.3389/fpls.2020.593938)
Supplement: Supplementary Figure 1 — PagPDS genomic sequences (exon 1–exon 5) from 84K (Populus alba × P. glandulosa). Blue lines indicate exons. Red lines represent the PAM sequence. Yellow highlights indicate the five target sites. Potri.014G148700 is PDS from P. trichocarpa. [file Data_Sheet_2.DOCX]

Supplementary Material


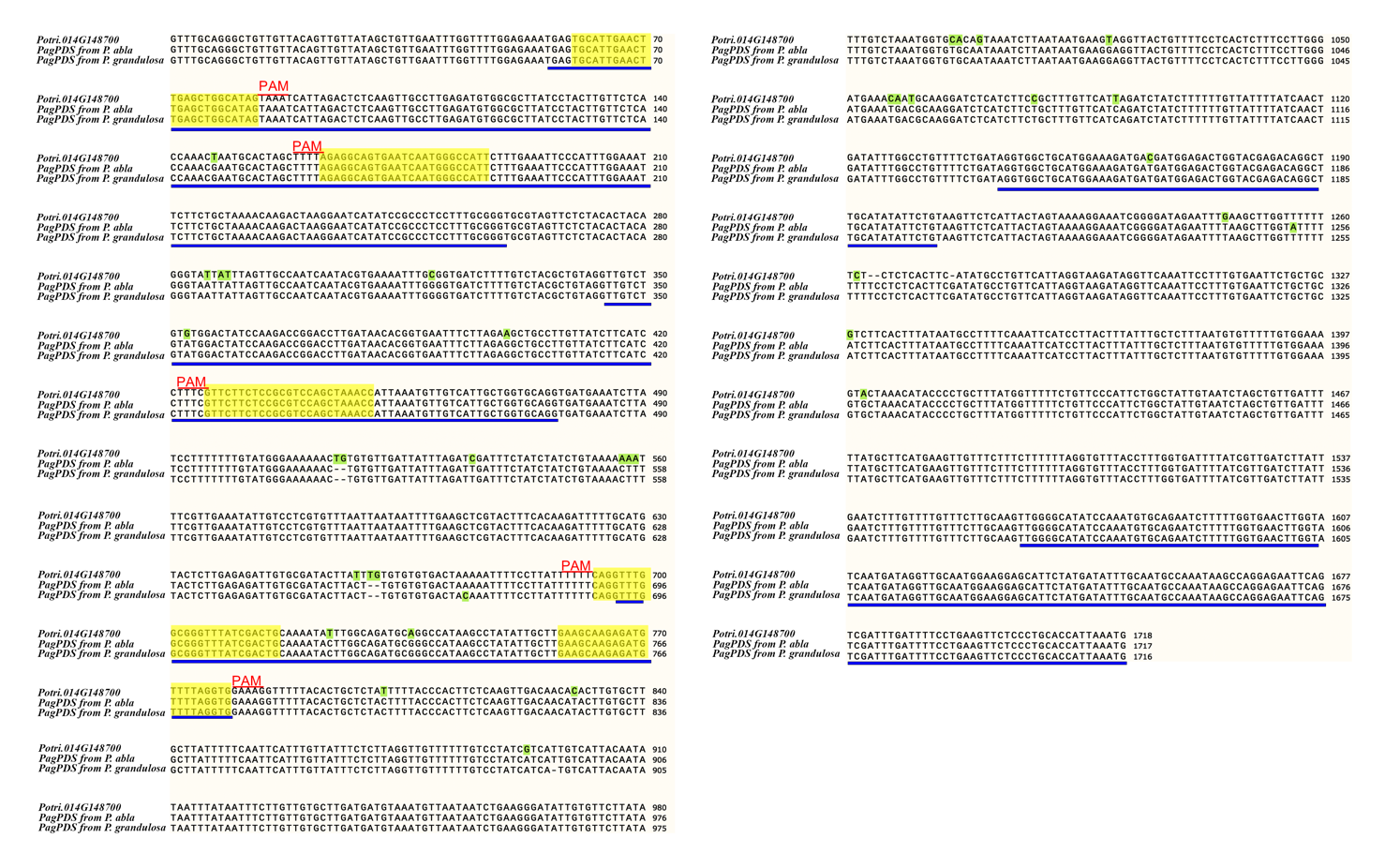


**Supplemental Figure 1:** *PagPDS* genomic sequences (exon1- exon5) from 84K (*Populus alba* × *P. glandulosa*). Blue lines indicate exons, the red lines represent PAM sequence, yellow highlighted indicate the 5 target sites. *Potri.014G148700* is *PDS* from *P. trichocarpa*.

**Supplemental sequence 1：**The crRNA expression cassette used in AsCas12a, LbCas12a and FnCas12a multiplex gene editing vectors. Letters in red indicate direct repeats (DR), highlighted indicate the 5 target sites, letters in blue indicate poly T.

>AtU6-DR-guide1-DR-guide2-DR-guide3-DR-guide4-DR-guide5-poly T

5’―GATCCAAGCTTCGTTGAACAACGGAAACTCGACTTGCCTTCCGCACAATACATCATTTCTTCTTAGCTTTTTTTCTTCTTCTTCGTTCATACAGTTTTTTTTTGTTTATCAGCTTACATTTTCTTGAACCGTAGCTTTCGTTTTCTTCTTTTTAACTTTCCATTCGGAGTTTTTGTATCTTGTTTCATAGTTTGTCCCAGGATTAGAATGATTAGGCATCGAACCTTCAAGAATTTGATTGAATAAAACATCTTCATTCTTAAGATATGAAGATAATCTTCAAAAGGCCCCTGGGAATCTGAAAGAAGAGAAGCAGGCCCATTTATATGGGAAAGAACAATAGTATTTCTTATATAGGCCCATTTAAGTTGAAAACAATCTTCAAAAGTCCCACATCGCTTAGATAAGAAAACGAAGCTGAGTTTATATACAGCTAGAGTCGAAGTAGTGATTGTAATTTCTACTAAGTGTAGATCTATGCCAGCTCAAGTTCAATGCATAATTTCTACTAAGTGTAGATGAGGCAGTGAATCAATGGGCCATTTAATTTCTACTAAGTGTAGATGTTCTTCTCCGCGTCCAGCTAAACTAATTTCTACTAAGTGTAGATCAGTCGATAAACCCGCCAAACCTGTAATTTCTACTAAGTGTAGATCACCTAAAACATCTCTTGCTTCAATTTTTTTGGTACC―3’
