# Supplementary material for: Efficient Genome Editing in Populus Using CRISPR/Cas12a
Source: Front Plant Sci. 2020 Nov 19;11:593938. doi: 10.3389/fpls.2020.593938 (PMC7720674; doi:10.3389/fpls.2020.593938)
Supplement: Supplementary Dataset 1 — LbCas12a- and FnCas12a-mediated gene editing in transgenic poplar plants. [file Data_Sheet_1.PDF]

**Dataset S1: LbCas12a and FnCas12a-mediated gene editing in transgenic poplar plants.**

**LbCas12a**

| line | phenotype | target1 | target2 | target3 | target4 | target5 |
|---|---|---|---|---|---|---|
| WT | | TGAGTGCATTGAACTTGAGCTGGCATAGTAAATCAT | AGCTTTTAGAGGCAGTGAATCAATGGGCCATTCTTT | ATCCTTTCGTTCTTCTCCGCGTCCAGCTAAACCATT | TATTTTTTCAGGTTTGGCGGGTTTATCGACTGCAAA | TTGCTTGAAGCAAGAGATGTTTTAGGTGGAAAGGTT |
| | | TGAGTGCATTGAACTTGAGCTGGCATAGTAAATCAT | AGCTTTTAGAGGCAGTGAATCAATGGGCCATTCTTT | ATCCTTTCGTTCTTCTCCGCGTCCAGCTAAACCATT | TATTTTTTCAGGTTTGGCGGGTTTATCGACTGCAAA | TTGCTTGAAGCAAGAGATGTTTTAGGTGGAAAGGTT |
| #1 | albinism | TGAGT-------------GCTGGCATAGTAAATCAT | AGCTTTTAGAGGCAGT--------------TTCTTT | ATCCTTTCGTTCTTCTCCGCGTCCAGCTAAACCATT | TATTTTTTCAGGTTTGGCGGGTTTATCGACTGCAAA | TTGCTTGAAGCAAGAGATGTTTTAGGTGGAAAGGTT |
| | | TGAGTGCATTGAACTTGAGCTGGCATAGTAAATCATT | AGCTTTTAGAGGCAGTGAATCA-------ATTCTTT | ATCCTTTCGTTCTTCTCCGCGTCCAGCTAAACCATT | TATTTTTTCAGGTTTGGCGGGTTTATCGACTGCAAA | TTGCTTGAAGCAAGAGATGTTTTAGGTGGAAAGGTT |
| #2 | albinism | TGAGT-------------GCTGGCATAGTAAATCAT | AGCTTTTAGAGGCAGT--------------TTCTTT | ATCCTTTCGTTCTTCTCCGCGTCCAGCTAAACCATT | TATTTTTTCAGGTTTGGCGGGTTTATCGACTGCAAA | TTGCTTGAAGCAAGAGATGTTTTAGGTGGAAAGGTT |
| | | TGAGT-------------GCTGGCATAGTAAATCAT | AGCTTTTAGAGGCAGT--------------TTCTTT | ATCCTTTCGTTCTTCTCCGCGTCCAGCTAAACCATT | TATTTTTTCAGGTTTGGCGGGTTTATCGACTGCAAA | TTGCTTGAAGCAAGAGATGTTTTAGGTGGAAAGGTT |
| #3 | albinism | TGAGT-------------GCTGGCATAGTAAATCAT | AGCTTTTAGAGGCAGTGAATCA-------ATTCTTT | ATCCTTTCGTTCTTCTCCGCGTCCAGCTAAACCATT | TATTTTTTCAGGTTTGGCGGGTTTATCGACTGCAAA | TTGCTTGAAGCAAGAGATGTTTTAGGTGGAAAGGTT |
| | | TGAGT-------------GCTGGCATAGTAAATCAT | AGCTTTTAGAGGCAGT--------------TTCTTT | ATCCTTTCGTTCTTCTCCGCGTCCAGCTAAACCATT | TATTTTTTCAGGTTTGGCGGGTTTATCGACTGCAAA | TTGCTTGAAGCAAGAGATGTTTTAGGTGGAAAGGTT |
| #4 | albinism | TGAGT-------------GCTGGCATAGTAAATCAT | AGCTTTTAGAGGCAGT--------------TTCTTT | ATCCTTTCGTTCTTCTCCGCGTCCAGCTAAACCATT | TATTTTTTCAGGTTTGGCGGGTTTATCGACTGCAAA | TTGCTTGAAGCAAGAGATGTTTTAGGTGGAAAGGTT |
| | | TGAGT-------------GCTGGCATAGTAAATCAT | AGCTTTTAGAGGCAGT--------------TTCTTT | ATCCTTTCGTTCTTCTCCGCGTCCAGCTAAACCATT | TATTTTTTCAGGTTTGGCGGGTTTATCGACTGCAAA | TTGCTTGAAGCAAGAGATGTTTTAGGTGGAAAGGTT |
| #5 | albinism | TGAGTGCATTGAACTTGAGCTGGCATAGTAAATCAT | AGCTTTTAGAGGCAGTGAATCA-------ATTCTTT | ATCCTTTCGTTCTTCTCCGCGTCCAGCTAAACCATT | TATTTTTTCAGGTTTGGCGGGTTTATCGACTGCAAA | TTGCTTGAAGCAAGAGATGTTTTAGGTGGAAAGGTT |
| | | TGAGT-------------GCTGGCATAGTAAATCAT | AGCTTTTAGAGGCAGT--------------TTCTTT | ATCCTTTCGTTCTTCTCCGCGTCCAGCTAAACCATT | TATTTTTTCAGGTTTGGCGGGTTTATCGACTGCAAA | TTGCTTGAAGCAAGAGATGTTTTAGGTGGAAAGGTT |
| #6 | pale | TGAGT------------------------------- | ------------------------------------ | ------------------------------------ | ----------------GCGGGTTTATCGACTGCAAA | TTGCTTGAAGCAAGAGATGTTTTAGGTGGAAAGGTT |
| | | TGAGTGCATTGAACTTGAGCTGGCATAGTAAATCAT | AGCTTTTAGAGGCAGTGAATCAATGGGCCATTCTTT | ATCCTTTCGTTCTTCTCCGCGTCCAGCTAAACCATT | TATTTTTTCAGGTTTGGCGGGTTTATCGACTGCAAA | TTGCTTGAAGCAAGAGATGTTTTAGGTGGAAAGGTT |
| #7 | pale | TGAGTGCATTGAACTTGAGCTGGCATAGTAAATCAT | AGCTTTTAGAGGCAGTGA------------------ | ------------------------------------ | ------------------------------------ | TTGCTTGAAGCAAGAGATGTTTTAGGTGGAAAGGTT |
| | | TGAGTGCATTGAACTTGAGCTGGCATAGTAAATCAT | AGCTTTTAGAGGCAGTGAATCAATGGGCCATTCTTT | ATCCTTTCGTTCTTCTCCGCGTCCAGCTAAACCATT | TATTTTTTCAGGTTTGGCGGGTTTATCGACTGCAAA | TTGCTTGAAGCAAGAGATGTTTTAGGTGGAAAGGTT |
| #8 | pale | TGAGTGCATTGAACTTGAGCTGGCATAGTAAATCAT | AGCTTTTAGAGGCAGTGAATCAATGGGCCATTCTTT | ATCCTTTCGTTCTTCTCCGCGTCCAGCTAAACCATT | TATTTTTTCAGGTTTGGCGGGTTTATCGACTGCAAA | TTGCTTGAAGCAAGAGATGTTTTAGGTGGAAAGGTT |
| | | TGAGTGCATTGAACTTGAGCTGGCATAGTAAATCAT | AGCTTTTAGAGGCAGTGAAT---------------- | -------------------------------CCGTT | TATTTTTTCAGGTTTGGCGGGTTTATCGACTGCAAA | TTGCTTGAAGCAAGAGATGTTTTAGGTGGAAAGGTT |
| #9 | pale | TGAGTGCATTGAACTTGAGCTGGCATAGTAAATCAT | AGCTTTTAGAGGCAGTGAATCAATGGGCCATTCTTT | ATCCTTTCGTTCTTCTCCGCGTCCAGCTAAACCATT | TATTTTTTCAGGTTTGGCGGGTTTATCGACTGCAAA | TTGCTTGAAGCAAGAGATGTTTTAGGTGGAAAGGTT |
| | | TGAGTGCATTGAACTTGAGCTGGCATAGTAAATCAT | AGCTTTTAGAGGCAGTGAAT---------------- | -------------------------------CCGTT | TATTTTTTCAGGTTTGGCGGGTTTATCGACTGCAAA | TTGCTTGAAGCAAGAGATGTTTTAGGTGGAAAGGTT |
| #10 | pale | TGAGTGCA-------TGAGCTGGCATAGTAAATCAT | AGCTTTTAGAGGCAGTGAA--------------TTT | ATCCTTTCGTTCTTCTCCGCGTCCAGCTAAACCATT | TATTTTTTCAGGTTTGGCGGGTTTATCGACTGCAAA | TTGCTTGAAGCAAGAGATGTTTTAGGTGGAAAGGTT |
| | | TGAGTGCATTGAACTTGAGCTGGCATAGTAAATCAT | AGCTTTTAGAGGCAGTGAATCAATGGGCCATTCTTT | ATCCTTTCGTTCTTCTCCGCGTCCAGCTAAACCATT | TATTTTTTCAGGTTTGGCGGGTTTATCGACTGCAAA | TTGCTTGAAGCAAGAGATGTTTTAGGTGGAAAGGTT |
| #11 | green | TGAGTGCATTGAACTTGAGCTGGCATAGTAAATCAT | AGCTTTTAGAGGCAGTGAATCAATGGGCCATTCTTT | ATCCTTTCGTTCTTCTCCGCGTCCAGCTAAACCATT | TATTTTTTCAGGTTTGGCGGGTTTATCGACTGCAAA | TTGCTTGAAGCAAGAGATGTTTTAGGTGGAAAGGTT |
| | | TGAGTGCATTGAACTTGAGCTGGCATAGTAAATCAT | AGCTTTTAGAGGCAGTGAATCAATGGGCCATTCTTT | ATCCTTTCGTTCTTCTCCGCGTCCAGCTAAACCATT | TATTTTTTCAGGTTTGGCGGGTTTATCGACTGCAAA | TTGCTTGAAGCAAGAGATGTTTTAGGTGGAAAGGTT |
| #12 | green | TGAGTGCATTGAACTTGAGCTGGCATAGTAAATCAT | AGCTTTTAGAGGCAGTGAATCAATGGGCCATTCTTT | ATCCTTTCGTTCTTCTCCGCGTCCAGCTAAACCATT | TATTTTTTCAGGTTTGGCGGGTTTATCGACTGCAAA | TTGCTTGAAGCAAGAGATGTTTTAGGTGGAAAGGTT |
| | | TGAGTGCATTGAACTTGAGCTGGCATAGTAAATCAT | AGCTTTTAGAGGCAGTGAATCAATGGGCCATTCTTT | ATCCTTTCGTTCTTCTCCGCGTCCAGCTAAACCATT | TATTTTTTCAGGTTTGGCGGGTTTATCGACTGCAAA | TTGCTTGAAGCAAGAGATGTTTTAGGTGGAAAGGTT |
| #13 | green | TGAGTGCATTGAACTTGAGCTGGCATAGTAAATCAT | AGCTTTTAGAGGCAGTGAATCAATGGGCCATTCTTT | ATCCTTTCGTTCTTCTCCGCGTCCAGCTAAACCATT | TATTTTTTCAGGTTTGGCGGGTTTATCGACTGCAAA | TTGCTTGAAGCAAGAGATGTTTTAGGTGGAAAGGTT |
| | | TGAGTGCATTGAACTTGAGCTGGCATAGTAAATCAT | AGCTTTTAGAGGCAGTGAATCAATGGGCCATTCTTT | ATCCTTTCGTTCTTCTCCGCGTCCAGCTAAACCATT | TATTTTTTCAGGTTTGGCGGGTTTATCGACTGCAAA | TTGCTTGAAGCAAGAGATGTTTTAGGTGGAAAGGTT |
| #14 | green | TGAGTGCATTGAACTTGAGCTGGCATAGTAAATCAT | AGCTTTTAGAGGCAGTGAATCAATGGGCCATTCTTT | ATCCTTTCGTTCTTCTCCGCGTCCAGCTAAACCATT | TATTTTTTCAGGTTTGGCGGGTTTATCGACTGCAAA | TTGCTTGAAGCAAGAGATGTTTTAGGTGGAAAGGTT |
| | | TGAGTGCATTGAACTTGAGCTGGCATAGTAAATCAT | AGCTTTTAGAGGCAGTGAATCAATGGGCCATTCTTT | ATCCTTTCGTTCTTCTCCGCGTCCAGCTAAACCATT | TATTTTTTCAGGTTTGGCGGGTTTATCGACTGCAAA | TTGCTTGAAGCAAGAGATGTTTTAGGTGGAAAGGTT |
| #15 | green | TGAGTGCATTGAACTTGAGCTGGCATAGTAAATCAT | AGCTTTTAGAGGCAGTGAATCAATGGGCCATTCTTT | ATCCTTTCGTTCTTCTCCGCGTCCAGCTAAACCATT | TATTTTTTCAGGTTTGGCGGGTTTATCGACTGCAAA | TTGCTTGAAGCAAGAGATGTTTTAGGTGGAAAGGTT |
| | | TGAGTGCATTGAACTTGAGCTGGCATAGTAAATCAT | AGCTTTTAGAGGCAGTGAATCAATGGGCCATTCTTT | ATCCTTTCGTTCTTCTCCGCGTCCAGCTAAACCATT | TATTTTTTCAGGTTTGGCGGGTTTATCGACTGCAAA | TTGCTTGAAGCAAGAGATGTTTTAGGTGGAAAGGTT |
| #16 | green | TGAGTGCATTGAACTTGAGCTGGCATAGTAAATCAT | AGCTTTTAGAGGCAGTGAATCAATGGGCCATTCTTT | ATCCTTTCGTTCTTCTCCGCGTCCAGCTAAACCATT | TATTTTTTCAGGTTTGGCGGGTTTATCGACTGCAAA | TTGCTTGAAGCAAGAGATGTTTTAGGTGGAAAGGTT |
| | | TGAGTGCATTGAACTTGAGCTGGCATAGTAAATCAT | AGCTTTTAGAGGCAGTGAATCAATGGGCCATTCTTT | ATCCTTTCGTTCTTCTCCGCGTCCAGCTAAACCATT | TATTTTTTCAGGTTTGGCGGGTTTATCGACTGCAAA | TTGCTTGAAGCAAGAGATGTTTTAGGTGGAAAGGTT |
| #17 | green | TGAGTGCATTGAACTTGAGCTGGCATAGTAAATCAT | AGCTTTTAGAGGCAGTGAATCAATGGGCCATTCTTT | ATCCTTTCGTTCTTCTCCGCGTCCAGCTAAACCATT | TATTTTTTCAGGTTTGGCGGGTTTATCGACTGCAAA | TTGCTTGAAGCAAGAGATGTTTTAGGTGGAAAGGTT |
| | | TGAGTGCATTGAACTTGAGCTGGCATAGTAAATCAT | AGCTTTTAGAGGCAGTGAATCAATGGGCCATTCTTT | ATCCTTTCGTTCTTCTCCGCGTCCAGCTAAACCATT | TATTTTTTCAGGTTTGGCGGGTTTATCGACTGCAAA | TTGCTTGAAGCAAGAGATGTTTTAGGTGGAAAGGTT |
| #18 | green | TGAGTGCATTGAACTTGAGCTGGCATAGTAAATCAT | AGCTTTTAGAGGCAGTGAATCAATGGGCCATTCTTT | ATCCTTTCGTTCTTCTCCGCGTCCAGCTAAACCATT | TATTTTTTCAGGTTTGGCGGGTTTATCGACTGCAAA | TTGCTTGAAGCAAGAGATGTTTTAGGTGGAAAGGTT |
| | | TGAGTGCATTGAACTTGAGCTGGCATAGTAAATCAT | AGCTTTTAGAGGCAGTGAATCAATGGGCCATTCTTT | ATCCTTTCGTTCTTCTCCGCGTCCAGCTAAACCATT | TATTTTTTCAGGTTTGGCGGGTTTATCGACTGCAAA | TTGCTTGAAGCAAGAGATGTTTTAGGTGGAAAGGTT |
| #19 | green | TGAGTGCATTGAACTTGAGCTGGCATAGTAAATCAT | AGCTTTTAGAGGCAGTGAATCAATGGGCCATTCTTT | ATCCTTTCGTTCTTCTCCGCGTCCAGCTAAACCATT | TATTTTTTCAGGTTTGGCGGGTTTATCGACTGCAAA | TTGCTTGAAGCAAGAGATGTTTTAGGTGGAAAGGTT |
| | | TGAGTGCATTGAACTTGAGCTGGCATAGTAAATCAT | AGCTTTTAGAGGCAGTGAATCAATGGGCCATTCTTT | ATCCTTTCGTTCTTCTCCGCGTCCAGCTAAACCATT | TATTTTTTCAGGTTTGGCGGGTTTATCGACTGCAAA | TTGCTTGAAGCAAGAGATGTTTTAGGTGGAAAGGTT |
| #20 | green | TGAGTGCATTGAACTTGAGCTGGCATAGTAAATCAT | AGCTTTTAGAGGCAGTGAATCAATGGGCCATTCTTT | ATCCTTTCGTTCTTCTCCGCGTCCAGCTAAACCATT | TATTTTTTCAGGTTTGGCGGGTTTATCGACTGCAAA | TTGCTTGAAGCAAGAGATGTTTTAGGTGGAAAGGTT |
| | | TGAGTGCATTGAACTTGAGCTGGCATAGTAAATCAT | AGCTTTTAGAGGCAGTGAATCAATGGGCCATTCTTT | ATCCTTTCGTTCTTCTCCGCGTCCAGCTAAACCATT | TATTTTTTCAGGTTTGGCGGGTTTATCGACTGCAAA | TTGCTTGAAGCAAGAGATGTTTTAGGTGGAAAGGTT |
| #21 | green | TGAGTGCATTGAACTTGAGCTGGCATAGTAAATCAT | AGCTTTTAGAGGCAGTGAATCAATGGGCCATTCTTT | ATCCTTTCGTTCTTCTCCGCGTCCAGCTAAACCATT | TATTTTTTCAGGTTTGGCGGGTTTATCGACTGCAAA | TTGCTTGAAGCAAGAGATGTTTTAGGTGGAAAGGTT |
| | | TGAGTGCATTGAACTTGAGCTGGCATAGTAAATCAT | AGCTTTTAGAGGCAGTGAATCAATGGGCCATTCTTT | ATCCTTTCGTTCTTCTCCGCGTCCAGCTAAACCATT | TATTTTTTCAGGTTTGGCGGGTTTATCGACTGCAAA | TTGCTTGAAGCAAGAGATGTTTTAGGTGGAAAGGTT |
| #22 | green | TGAGTGCATTGAACTTGAGCTGGCATAGTAAATCAT | AGCTTTTAGAGGCAGTGAATCAATGGGCCATTCTTT | ATCCTTTCGTTCTTCTCCGCGTCCAGCTAAACCATT | TATTTTTTCAGGTTTGGCGGGTTTATCGACTGCAAA | TTGCTTGAAGCAAGAGATGTTTTAGGTGGAAAGGTT |
| | | TGAGTGCATTGAACTTGAGCTGGCATAGTAAATCAT | AGCTTTTAGAGGCAGTGAATCAATGGGCCATTCTTT | ATCCTTTCGTTCTTCTCCGCGTCCAGCTAAACCATT | TATTTTTTCAGGTTTGGCGGGTTTATCGACTGCAAA | TTGCTTGAAGCAAGAGATGTTTTAGGTGGAAAGGTT |
| #23 | green | TGAGTGCATTGAACTTGAGCTGGCATAGTAAATCAT | AGCTTTTAGAGGCAGTGAATCAATGGGCCATTCTTT | ATCCTTTCGTTCTTCTCCGCGTCCAGCTAAACCATT | TATTTTTTCAGGTTTGGCGGGTTTATCGACTGCAAA | TTGCTTGAAGCAAGAGATGTTTTAGGTGGAAAGGTT |
| | | TGAGTGCATTGAACTTGAGCTGGCATAGTAAATCAT | AGCTTTTAGAGGCAGTGAATCAATGGGCCATTCTTT | ATCCTTTCGTTCTTCTCCGCGTCCAGCTAAACCATT | TATTTTTTCAGGTTTGGCGGGTTTATCGACTGCAAA | TTGCTTGAAGCAAGAGATGTTTTAGGTGGAAAGGTT |
| #24 | green | TGAGTGCATTGAACTTGAGCTGGCATAGTAAATCAT | AGCTTTTAGAGGCAGTGAATCAATGGGCCATTCTTT | ATCCTTTCGTTCTTCTCCGCGTCCAGCTAAACCATT | TATTTTTTCAGGTTTGGCGGGTTTATCGACTGCAAA | TTGCTTGAAGCAAGAGATGTTTTAGGTGGAAAGGTT |
| | | TGAGTGCATTGAACTTGAGCTGGCATAGTAAATCAT | AGCTTTTAGAGGCAGTGAATCAATGGGCCATTCTTT | ATCCTTTCGTTCTTCTCCGCGTCCAGCTAAACCATT | TATTTTTTCAGGTTTGGCGGGTTTATCGACTGCAAA | TTGCTTGAAGCAAGAGATGTTTTAGGTGGAAAGGTT |
| #25 | green | TGAGTGCATTGAACTTGAGCTGGCATAGTAAATCAT | AGCTTTTAGAGGCAGTGAATCAATGGGCCATTCTTT | ATCCTTTCGTTCTTCTCCGCGTCCAGCTAAACCATT | TATTTTTTCAGGTTTGGCGGGTTTATCGACTGCAAA | TTGCTTGAAGCAAGAGATGTTTTAGGTGGAAAGGTT |
| | | TGAGTGCATTGAACTTGAGCTGGCATAGTAAATCAT | AGCTTTTAGAGGCAGTGAATCAATGGGCCATTCTTT | ATCCTTTCGTTCTTCTCCGCGTCCAGCTAAACCATT | TATTTTTTCAGGTTTGGCGGGTTTATCGACTGCAAA | TTGCTTGAAGCAAGAGATGTTTTAGGTGGAAAGGTT |
| #26 | green | TGAGTGCATTGAACTTGAGCTGGCATAGTAAATCAT | AGCTTTTAGAGGCAGTGAATCAATGGGCCATTCTTT | ATCCTTTCGTTCTTCTCCGCGTCCAGCTAAACCATT | TATTTTTTCAGGTTTGGCGGGTTTATCGACTGCAAA | TTGCTTGAAGCAAGAGATGTTTTAGGTGGAAAGGTT |
| | | TGAGTGCATTGAACTTGAGCTGGCATAGTAAATCAT | AGCTTTTAGAGGCAGTGAATCAATGGGCCATTCTTT | ATCCTTTCGTTCTTCTCCGCGTCCAGCTAAACCATT | TATTTTTTCAGGTTTGGCGGGTTTATCGACTGCAAA | TTGCTTGAAGCAAGAGATGTTTTAGGTGGAAAGGTT |
| #27 | green | TGAGTGCATTGAACTTGAGCTGGCATAGTAAATCAT | AGCTTTTAGAGGCAGTGAATCAATGGGCCATTCTTT | ATCCTTTCGTTCTTCTCCGCGTCCAGCTAAACCATT | TATTTTTTCAGGTTTGGCGGGTTTATCGACTGCAAA | TTGCTTGAAGCAAGAGATGTTTTAGGTGGAAAGGTT |
| | | TGAGTGCATTGAACTTGAGCTGGCATAGTAAATCAT | AGCTTTTAGAGGCAGTGAATCAATGGGCCATTCTTT | ATCCTTTCGTTCTTCTCCGCGTCCAGCTAAACCATT | TATTTTTTCAGGTTTGGCGGGTTTATCGACTGCAAA | TTGCTTGAAGCAAGAGATGTTTTAGGTGGAAAGGTT |
| #28 | green | TGAGTGCATTGAACTTGAGCTGGCATAGTAAATCAT | AGCTTTTAGAGGCAGTGAATCAATGGGCCATTCTTT | ATCCTTTCGTTCTTCTCCGCGTCCAGCTAAACCATT | TATTTTTTCAGGTTTGGCGGGTTTATCGACTGCAAA | TTGCTTGAAGCAAGAGATGTTTTAGGTGGAAAGGTT |
| | | TGAGTGCATTGAACTTGAGCTGGCATAGTAAATCAT | AGCTTTTAGAGGCAGTGAATCAATGGGCCATTCTTT | ATCCTTTCGTTCTTCTCCGCGTCCAGCTAAACCATT | TATTTTTTCAGGTTTGGCGGGTTTATCGACTGCAAA | TTGCTTGAAGCAAGAGATGTTTTAGGTGGAAAGGTT |
| #29 | green | TGAGTGCATTGAACTTGAGCTGGCATAGTAAATCAT | AGCTTTTAGAGGCAGTGAATCAATGGGCCATTCTTT | ATCCTTTCGTTCTTCTCCGCGTCCAGCTAAACCATT | TATTTTTTCAGGTTTGGCGGGTTTATCGACTGCAAA | TTGCTTGAAGCAAGAGATGTTTTAGGTGGAAAGGTT |
| | | TGAGTGCATTGAACTTGAGCTGGCATAGTAAATCAT | AGCTTTTAGAGGCAGTGAATCAATGGGCCATTCTTT | ATCCTTTCGTTCTTCTCCGCGTCCAGCTAAACCATT | TATTTTTTCAGGTTTGGCGGGTTTATCGACTGCAAA | TTGCTTGAAGCAAGAGATGTTTTAGGTGGAAAGGTT |
| #30 | green | TGAGTGCATTGAACTTGAGCTGGCATAGTAAATCAT | AGCTTTTAGAGGCAGTGAATCAATGGGCCATTCTTT | ATCCTTTCGTTCTTCTCCGCGTCCAGCTAAACCATT | TATTTTTTCAGGTTTGGCGGGTTTATCGACTGCAAA | TTGCTTGAAGCAAGAGATGTTTTAGGTGGAAAGGTT |
| | | TGAGTGCATTGAACTTGAGCTGGCATAGTAAATCAT | AGCTTTTAGAGGCAGTGAATCAATGGGCCATTCTTT | ATCCTTTCGTTCTTCTCCGCGTCCAGCTAAACCATT | TATTTTTTCAGGTTTGGCGGGTTTATCGACTGCAAA | TTGCTTGAAGCAAGAGATGTTTTAGGTGGAAAGGTT |

## FnCas12a

| line | phenotype | target1 | target2 | target3 | target4 | target5 |
|---|---|---|---|---|---|---|
| WT | | TGAGTGCATTGAACTTGAGCTGGCATAGTAAATCAT | AGCTTTTAGAGGCAGTGAATCAATGGGCCATTCTTT | ATCCTTTCGTTCTTCTCCGCGTCCAGCTAAACCATT | TATTTTTTCAGGTTTGGCGGGTTTATCGACTGCAAA | TTGCTTGAAGCAAGAGATGTTTTAGGTGGAAAGGTT |
| | | TGAGTGCATTGAACTTGAGCTGGCATAGTAAATCAT | AGCTTTTAGAGGCAGTGAATCAATGGGCCATTCTTT | ATCCTTTCGTTCTTCTCCGCGTCCAGCTAAACCATT | TATTTTTTCAGGTTTGGCGGGTTTATCGACTGCAAA | TTGCTTGAAGCAAGAGATGTTTTAGGTGGAAAGGTT |
| #1 | pale | TGAGTGCA-------TGAGCTGGCATAGTAAATCAT | AGCTTTTAGAGGCAGTGAA---------------TT | ATCCTTTCGTTCTTCTCCGCGTCCAGCTAAACCATT | TATTTTTTCAGGTTTGGCGGGTTTATCGACTGCAAA | TTGCTTGAAGCAAGAGATGTTTTAGGTGGAAAGGTT |
| | | TGAGTGCATTGAACTTGAGCTGGCATAGTAAATCAT | AGCTTTTAGAGGCAGTGAATCAATGGGCCATTCTTT | ATCCTTTCGTTCTTCTCCGCGTCCAGCTAAACCATT | TATTTTTTCAGGTTTGGCGGGTTTATCGACTGCAAA | TTGCTTGAAGCAAGAGATGTTTTAGGTGGAAAGGTT |
| #2 | pale | TGAGTGCA-------TGAGCTGGCATAGTAAATCAT | AGCTTTTAGAGGCAGTGAA---------------TT | ATCCTTTCGTTCTTCTCCGCGTCCAGCTAAACCATT | TATTTTTTCAGGTTTGGCGGGTTTATCGACTGCAAA | TTGCTTGAAGCAAGAGATGTTTTAGGTGGAAAGGTT |
| | | TGAGTGCATTGAACTTGAGCTGGCATAGTAAATCAT | AGCTTTTAGAGGCAGTGAATCAATGGGCCATTCTTT | ATCCTTTCGTTCTTCTCCGCGTCCAGCTAAACCATT | TATTTTTTCAGGTTTGGCGGGTTTATCGACTGCAAA | TTGCTTGAAGCAAGAGATGTTTTAGGTGGAAAGGTT |
| #3 | green | TGAGTGCATTGAACTTGAGCTGGCATAGTAAATCAT | AGCTTTTAGAGGCAGTGAATCAATGGGCCATTCTTT | ATCCTTTCGTTCTTCTCCGCGTCCAGCTAAACCATT | TATTTTTTCAGGTTTGGCGGGTTTATCGACTGCAAA | TTGCTTGAAGCAAGAGATGTTTTAGGTGGAAAGGTT |
| | | TGAGTGCATTGAACTTGAGCTGGCATAGTAAATCAT | AGCTTTTAGAGGCAGTGAATCAATGGGCCATTCTTT | ATCCTTTCGTTCTTCTCCGCGTCCAGCTAAACCATT | TATTTTTTCAGGTTTGGCGGGTTTATCGACTGCAAA | TTGCTTGAAGCAAGAGATGTTTTAGGTGGAAAGGTT |
| #4 | green | TGAGTGCATTGAACTTGAGCTGGCATAGTAAATCAT | AGCTTTTAGAGGCAGTGAATCAATGGGCCATTCTTT | ATCCTTTCGTTCTTCTCCGCGTCCAGCTAAACCATT | TATTTTTTCAGGTTTGGCGGGTTTATCGACTGCAAA | TTGCTTGAAGCAAGAGATGTTTTAGGTGGAAAGGTT |
| | | TGAGTGCATTGAACTTGAGCTGGCATAGTAAATCAT | AGCTTTTAGAGGCAGTGAATCAATGGGCCATTCTTT | ATCCTTTCGTTCTTCTCCGCGTCCAGCTAAACCATT | TATTTTTTCAGGTTTGGCGGGTTTATCGACTGCAAA | TTGCTTGAAGCAAGAGATGTTTTAGGTGGAAAGGTT |
| #5 | green | TGAGTGCATTGAACTTGAGCTGGCATAGTAAATCAT | AGCTTTTAGAGGCAGTGAATCAATGGGCCATTCTTT | ATCCTTTCGTTCTTCTCCGCGTCCAGCTAAACCATT | TATTTTTTCAGGTTTGGCGGGTTTATCGACTGCAAA | TTGCTTGAAGCAAGAGATGTTTTAGGTGGAAAGGTT |
| | | TGAGTGCATTGAACTTGAGCTGGCATAGTAAATCAT | AGCTTTTAGAGGCAGTGAATCAATGGGCCATTCTTT | ATCCTTTCGTTCTTCTCCGCGTCCAGCTAAACCATT | TATTTTTTCAGGTTTGGCGGGTTTATCGACTGCAAA | TTGCTTGAAGCAAGAGATGTTTTAGGTGGAAAGGTT |
| #6 | green | TGAGTGCATTGAACTTGAGCTGGCATAGTAAATCAT | AGCTTTTAGAGGCAGTGAATCAATGGGCCATTCTTT | ATCCTTTCGTTCTTCTCCGCGTCCAGCTAAACCATT | TATTTTTTCAGGTTTGGCGGGTTTATCGACTGCAAA | TTGCTTGAAGCAAGAGATGTTTTAGGTGGAAAGGTT |
| | | TGAGTGCATTGAACTTGAGCTGGCATAGTAAATCAT | AGCTTTTAGAGGCAGTGAATCAATGGGCCATTCTTT | ATCCTTTCGTTCTTCTCCGCGTCCAGCTAAACCATT | TATTTTTTCAGGTTTGGCGGGTTTATCGACTGCAAA | TTGCTTGAAGCAAGAGATGTTTTAGGTGGAAAGGTT |
| #7 | green | TGAGTGCATTGAACTTGAGCTGGCATAGTAAATCAT | AGCTTTTAGAGGCAGTGAATCAATGGGCCATTCTTT | ATCCTTTCGTTCTTCTCCGCGTCCAGCTAAACCATT | TATTTTTTCAGGTTTGGCGGGTTTATCGACTGCAAA | TTGCTTGAAGCAAGAGATGTTTTAGGTGGAAAGGTT |
| | | TGAGTGCATTGAACTTGAGCTGGCATAGTAAATCAT | AGCTTTTAGAGGCAGTGAATCAATGGGCCATTCTTT | ATCCTTTCGTTCTTCTCCGCGTCCAGCTAAACCATT | TATTTTTTCAGGTTTGGCGGGTTTATCGACTGCAAA | TTGCTTGAAGCAAGAGATGTTTTAGGTGGAAAGGTT |
| #8 | green | TGAGTGCATTGAACTTGAGCTGGCATAGTAAATCAT | AGCTTTTAGAGGCAGTGAATCAATGGGCCATTCTTT | ATCCTTTCGTTCTTCTCCGCGTCCAGCTAAACCATT | TATTTTTTCAGGTTTGGCGGGTTTATCGACTGCAAA | TTGCTTGAAGCAAGAGATGTTTTAGGTGGAAAGGTT |
| | | TGAGTGCATTGAACTTGAGCTGGCATAGTAAATCAT | AGCTTTTAGAGGCAGTGAATCAATGGGCCATTCTTT | ATCCTTTCGTTCTTCTCCGCGTCCAGCTAAACCATT | TATTTTTTCAGGTTTGGCGGGTTTATCGACTGCAAA | TTGCTTGAAGCAAGAGATGTTTTAGGTGGAAAGGTT |
| #9 | green | TGAGTGCATTGAACTTGAGCTGGCATAGTAAATCAT | AGCTTTTAGAGGCAGTGAATCAATGGGCCATTCTTT | ATCCTTTCGTTCTTCTCCGCGTCCAGCTAAACCATT | TATTTTTTCAGGTTTGGCGGGTTTATCGACTGCAAA | TTGCTTGAAGCAAGAGATGTTTTAGGTGGAAAGGTT |
| | | TGAGTGCATTGAACTTGAGCTGGCATAGTAAATCAT | AGCTTTTAGAGGCAGTGAATCAATGGGCCATTCTTT | ATCCTTTCGTTCTTCTCCGCGTCCAGCTAAACCATT | TATTTTTTCAGGTTTGGCGGGTTTATCGACTGCAAA | TTGCTTGAAGCAAGAGATGTTTTAGGTGGAAAGGTT |
| #10 | green | TGAGTGCATTGAACTTGAGCTGGCATAGTAAATCAT | AGCTTTTAGAGGCAGTGAATCAATGGGCCATTCTTT | ATCCTTTCGTTCTTCTCCGCGTCCAGCTAAACCATT | TATTTTTTCAGGTTTGGCGGGTTTATCGACTGCAAA | TTGCTTGAAGCAAGAGATGTTTTAGGTGGAAAGGTT |
| | | TGAGTGCATTGAACTTGAGCTGGCATAGTAAATCAT | AGCTTTTAGAGGCAGTGAATCAATGGGCCATTCTTT | ATCCTTTCGTTCTTCTCCGCGTCCAGCTAAACCATT | TATTTTTTCAGGTTTGGCGGGTTTATCGACTGCAAA | TTGCTTGAAGCAAGAGATGTTTTAGGTGGAAAGGTT |
| #11 | green | TGAGTGCATTGAACTTGAGCTGGCATAGTAAATCAT | AGCTTTTAGAGGCAGTGAATCAATGGGCCATTCTTT | ATCCTTTCGTTCTTCTCCGCGTCCAGCTAAACCATT | TATTTTTTCAGGTTTGGCGGGTTTATCGACTGCAAA | TTGCTTGAAGCAAGAGATGTTTTAGGTGGAAAGGTT |
| | | TGAGTGCATTGAACTTGAGCTGGCATAGTAAATCAT | AGCTTTTAGAGGCAGTGAATCAATGGGCCATTCTTT | ATCCTTTCGTTCTTCTCCGCGTCCAGCTAAACCATT | TATTTTTTCAGGTTTGGCGGGTTTATCGACTGCAAA | TTGCTTGAAGCAAGAGATGTTTTAGGTGGAAAGGTT |
| #12 | green | TGAGTGCATTGAACTTGAGCTGGCATAGTAAATCAT | AGCTTTTAGAGGCAGTGAATCAATGGGCCATTCTTT | ATCCTTTCGTTCTTCTCCGCGTCCAGCTAAACCATT | TATTTTTTCAGGTTTGGCGGGTTTATCGACTGCAAA | TTGCTTGAAGCAAGAGATGTTTTAGGTGGAAAGGTT |
| | | TGAGTGCATTGAACTTGAGCTGGCATAGTAAATCAT | AGCTTTTAGAGGCAGTGAATCAATGGGCCATTCTTT | ATCCTTTCGTTCTTCTCCGCGTCCAGCTAAACCATT | TATTTTTTCAGGTTTGGCGGGTTTATCGACTGCAAA | TTGCTTGAAGCAAGAGATGTTTTAGGTGGAAAGGTT |
| #13 | green | TGAGTGCATTGAACTTGAGCTGGCATAGTAAATCAT | AGCTTTTAGAGGCAGTGAATCAATGGGCCATTCTTT | ATCCTTTCGTTCTTCTCCGCGTCCAGCTAAACCATT | TATTTTTTCAGGTTTGGCGGGTTTATCGACTGCAAA | TTGCTTGAAGCAAGAGATGTTTTAGGTGGAAAGGTT |
| | | TGAGTGCATTGAACTTGAGCTGGCATAGTAAATCAT | AGCTTTTAGAGGCAGTGAATCAATGGGCCATTCTTT | ATCCTTTCGTTCTTCTCCGCGTCCAGCTAAACCATT | TATTTTTTCAGGTTTGGCGGGTTTATCGACTGCAAA | TTGCTTGAAGCAAGAGATGTTTTAGGTGGAAAGGTT |
| #14 | green | TGAGTGCATTGAACTTGAGCTGGCATAGTAAATCAT | AGCTTTTAGAGGCAGTGAATCAATGGGCCATTCTTT | ATCCTTTCGTTCTTCTCCGCGTCCAGCTAAACCATT | TATTTTTTCAGGTTTGGCGGGTTTATCGACTGCAAA | TTGCTTGAAGCAAGAGATGTTTTAGGTGGAAAGGTT |
| | | TGAGTGCATTGAACTTGAGCTGGCATAGTAAATCAT | AGCTTTTAGAGGCAGTGAATCAATGGGCCATTCTTT | ATCCTTTCGTTCTTCTCCGCGTCCAGCTAAACCATT | TATTTTTTCAGGTTTGGCGGGTTTATCGACTGCAAA | TTGCTTGAAGCAAGAGATGTTTTAGGTGGAAAGGTT |
| #15 | green | TGAGTGCATTGAACTTGAGCTGGCATAGTAAATCAT | AGCTTTTAGAGGCAGTGAATCAATGGGCCATTCTTT | ATCCTTTCGTTCTTCTCCGCGTCCAGCTAAACCATT | TATTTTTTCAGGTTTGGCGGGTTTATCGACTGCAAA | TTGCTTGAAGCAAGAGATGTTTTAGGTGGAAAGGTT |
| | | TGAGTGCATTGAACTTGAGCTGGCATAGTAAATCAT | AGCTTTTAGAGGCAGTGAATCAATGGGCCATTCTTT | ATCCTTTCGTTCTTCTCCGCGTCCAGCTAAACCATT | TATTTTTTCAGGTTTGGCGGGTTTATCGACTGCAAA | TTGCTTGAAGCAAGAGATGTTTTAGGTGGAAAGGTT |
| #16 | green | TGAGTGCATTGAACTTGAGCTGGCATAGTAAATCAT | AGCTTTTAGAGGCAGTGAATCAATGGGCCATTCTTT | ATCCTTTCGTTCTTCTCCGCGTCCAGCTAAACCATT | TATTTTTTCAGGTTTGGCGGGTTTATCGACTGCAAA | TTGCTTGAAGCAAGAGATGTTTTAGGTGGAAAGGTT |
| | | TGAGTGCATTGAACTTGAGCTGGCATAGTAAATCAT | AGCTTTTAGAGGCAGTGAATCAATGGGCCATTCTTT | ATCCTTTCGTTCTTCTCCGCGTCCAGCTAAACCATT | TATTTTTTCAGGTTTGGCGGGTTTATCGACTGCAAA | TTGCTTGAAGCAAGAGATGTTTTAGGTGGAAAGGTT |
| #17 | green | TGAGTGCATTGAACTTGAGCTGGCATAGTAAATCAT | AGCTTTTAGAGGCAGTGAATCAATGGGCCATTCTTT | ATCCTTTCGTTCTTCTCCGCGTCCAGCTAAACCATT | TATTTTTTCAGGTTTGGCGGGTTTATCGACTGCAAA | TTGCTTGAAGCAAGAGATGTTTTAGGTGGAAAGGTT |
| | | TGAGTGCATTGAACTTGAGCTGGCATAGTAAATCAT | AGCTTTTAGAGGCAGTGAATCAATGGGCCATTCTTT | ATCCTTTCGTTCTTCTCCGCGTCCAGCTAAACCATT | TATTTTTTCAGGTTTGGCGGGTTTATCGACTGCAAA | TTGCTTGAAGCAAGAGATGTTTTAGGTGGAAAGGTT |
| #18 | green | TGAGTGCATTGAACTTGAGCTGGCATAGTAAATCAT | AGCTTTTAGAGGCAGTGAATCAATGGGCCATTCTTT | ATCCTTTCGTTCTTCTCCGCGTCCAGCTAAACCATT | TATTTTTTCAGGTTTGGCGGGTTTATCGACTGCAAA | TTGCTTGAAGCAAGAGATGTTTTAGGTGGAAAGGTT |
| | | TGAGTGCATTGAACTTGAGCTGGCATAGTAAATCAT | AGCTTTTAGAGGCAGTGAATCAATGGGCCATTCTTT | ATCCTTTCGTTCTTCTCCGCGTCCAGCTAAACCATT | TATTTTTTCAGGTTTGGCGGGTTTATCGACTGCAAA | TTGCTTGAAGCAAGAGATGTTTTAGGTGGAAAGGTT |
| #19 | green | TGAGTGCATTGAACTTGAGCTGGCATAGTAAATCAT | AGCTTTTAGAGGCAGTGAATCAATGGGCCATTCTTT | ATCCTTTCGTTCTTCTCCGCGTCCAGCTAAACCATT | TATTTTTTCAGGTTTGGCGGGTTTATCGACTGCAAA | TTGCTTGAAGCAAGAGATGTTTTAGGTGGAAAGGTT |
| | | TGAGTGCATTGAACTTGAGCTGGCATAGTAAATCAT | AGCTTTTAGAGGCAGTGAATCAATGGGCCATTCTTT | ATCCTTTCGTTCTTCTCCGCGTCCAGCTAAACCATT | TATTTTTTCAGGTTTGGCGGGTTTATCGACTGCAAA | TTGCTTGAAGCAAGAGATGTTTTAGGTGGAAAGGTT |
| #20 | green | TGAGTGCATTGAACTTGAGCTGGCATAGTAAATCAT | AGCTTTTAGAGGCAGTGAATCAATGGGCCATTCTTT | ATCCTTTCGTTCTTCTCCGCGTCCAGCTAAACCATT | TATTTTTTCAGGTTTGGCGGGTTTATCGACTGCAAA | TTGCTTGAAGCAAGAGATGTTTTAGGTGGAAAGGTT |
| | | TGAGTGCATTGAACTTGAGCTGGCATAGTAAATCAT | AGCTTTTAGAGGCAGTGAATCAATGGGCCATTCTTT | ATCCTTTCGTTCTTCTCCGCGTCCAGCTAAACCATT | TATTTTTTCAGGTTTGGCGGGTTTATCGACTGCAAA | TTGCTTGAAGCAAGAGATGTTTTAGGTGGAAAGGTT |
| #21 | green | TGAGTGCATTGAACTTGAGCTGGCATAGTAAATCAT | AGCTTTTAGAGGCAGTGAATCAATGGGCCATTCTTT | ATCCTTTCGTTCTTCTCCGCGTCCAGCTAAACCATT | TATTTTTTCAGGTTTGGCGGGTTTATCGACTGCAAA | TTGCTTGAAGCAAGAGATGTTTTAGGTGGAAAGGTT |
| | | TGAGTGCATTGAACTTGAGCTGGCATAGTAAATCAT | AGCTTTTAGAGGCAGTGAATCAATGGGCCATTCTTT | ATCCTTTCGTTCTTCTCCGCGTCCAGCTAAACCATT | TATTTTTTCAGGTTTGGCGGGTTTATCGACTGCAAA | TTGCTTGAAGCAAGAGATGTTTTAGGTGGAAAGGTT |
| #22 | green | TGAGTGCATTGAACTTGAGCTGGCATAGTAAATCAT | AGCTTTTAGAGGCAGTGAATCAATGGGCCATTCTTT | ATCCTTTCGTTCTTCTCCGCGTCCAGCTAAACCATT | TATTTTTTCAGGTTTGGCGGGTTTATCGACTGCAAA | TTGCTTGAAGCAAGAGATGTTTTAGGTGGAAAGGTT |
| | | TGAGTGCATTGAACTTGAGCTGGCATAGTAAATCAT | AGCTTTTAGAGGCAGTGAATCAATGGGCCATTCTTT | ATCCTTTCGTTCTTCTCCGCGTCCAGCTAAACCATT | TATTTTTTCAGGTTTGGCGGGTTTATCGACTGCAAA | TTGCTTGAAGCAAGAGATGTTTTAGGTGGAAAGGTT |
| #23 | green | TGAGTGCATTGAACTTGAGCTGGCATAGTAAATCAT | AGCTTTTAGAGGCAGTGAATCAATGGGCCATTCTTT | ATCCTTTCGTTCTTCTCCGCGTCCAGCTAAACCATT | TATTTTTTCAGGTTTGGCGGGTTTATCGACTGCAAA | TTGCTTGAAGCAAGAGATGTTTTAGGTGGAAAGGTT |
| | | TGAGTGCATTGAACTTGAGCTGGCATAGTAAATCAT | AGCTTTTAGAGGCAGTGAATCAATGGGCCATTCTTT | ATCCTTTCGTTCTTCTCCGCGTCCAGCTAAACCATT | TATTTTTTCAGGTTTGGCGGGTTTATCGACTGCAAA | TTGCTTGAAGCAAGAGATGTTTTAGGTGGAAAGGTT |
| #24 | green | TGAGTGCATTGAACTTGAGCTGGCATAGTAAATCAT | AGCTTTTAGAGGCAGTGAATCAATGGGCCATTCTTT | ATCCTTTCGTTCTTCTCCGCGTCCAGCTAAACCATT | TATTTTTTCAGGTTTGGCGGGTTTATCGACTGCAAA | TTGCTTGAAGCAAGAGATGTTTTAGGTGGAAAGGTT |
| | | TGAGTGCATTGAACTTGAGCTGGCATAGTAAATCAT | AGCTTTTAGAGGCAGTGAATCAATGGGCCATTCTTT | ATCCTTTCGTTCTTCTCCGCGTCCAGCTAAACCATT | TATTTTTTCAGGTTTGGCGGGTTTATCGACTGCAAA | TTGCTTGAAGCAAGAGATGTTTTAGGTGGAAAGGTT |
| #25 | green | TGAGTGCATTGAACTTGAGCTGGCATAGTAAATCAT | AGCTTTTAGAGGCAGTGAATCAATGGGCCATTCTTT | ATCCTTTCGTTCTTCTCCGCGTCCAGCTAAACCATT | TATTTTTTCAGGTTTGGCGGGTTTATCGACTGCAAA | TTGCTTGAAGCAAGAGATGTTTTAGGTGGAAAGGTT |
| | | TGAGTGCATTGAACTTGAGCTGGCATAGTAAATCAT | AGCTTTTAGAGGCAGTGAATCAATGGGCCATTCTTT | ATCCTTTCGTTCTTCTCCGCGTCCAGCTAAACCATT | TATTTTTTCAGGTTTGGCGGGTTTATCGACTGCAAA | TTGCTTGAAGCAAGAGATGTTTTAGGTGGAAAGGTT |
| #26 | green | TGAGTGCATTGAACTTGAGCTGGCATAGTAAATCAT | AGCTTTTAGAGGCAGTGAATCAATGGGCCATTCTTT | ATCCTTTCGTTCTTCTCCGCGTCCAGCTAAACCATT | TATTTTTTCAGGTTTGGCGGGTTTATCGACTGCAAA | TTGCTTGAAGCAAGAGATGTTTTAGGTGGAAAGGTT |
| | | TGAGTGCATTGAACTTGAGCTGGCATAGTAAATCAT | AGCTTTTAGAGGCAGTGAATCAATGGGCCATTCTTT | ATCCTTTCGTTCTTCTCCGCGTCCAGCTAAACCATT | TATTTTTTCAGGTTTGGCGGGTTTATCGACTGCAAA | TTGCTTGAAGCAAGAGATGTTTTAGGTGGAAAGGTT |
| #27 | green | TGAGTGCATTGAACTTGAGCTGGCATAGTAAATCAT | AGCTTTTAGAGGCAGTGAATCAATGGGCCATTCTTT | ATCCTTTCGTTCTTCTCCGCGTCCAGCTAAACCATT | TATTTTTTCAGGTTTGGCGGGTTTATCGACTGCAAA | TTGCTTGAAGCAAGAGATGTTTTAGGTGGAAAGGTT |
| | | TGAGTGCATTGAACTTGAGCTGGCATAGTAAATCAT | AGCTTTTAGAGGCAGTGAATCAATGGGCCATTCTTT | ATCCTTTCGTTCTTCTCCGCGTCCAGCTAAACCATT | TATTTTTTCAGGTTTGGCGGGTTTATCGACTGCAAA | TTGCTTGAAGCAAGAGATGTTTTAGGTGGAAAGGTT |
| #28 | green | TGAGTGCATTGAACTTGAGCTGGCATAGTAAATCAT | AGCTTTTAGAGGCAGTGAATCAATGGGCCATTCTTT | ATCCTTTCGTTCTTCTCCGCGTCCAGCTAAACCATT | TATTTTTTCAGGTTTGGCGGGTTTATCGACTGCAAA | TTGCTTGAAGCAAGAGATGTTTTAGGTGGAAAGGTT |
| | | TGAGTGCATTGAACTTGAGCTGGCATAGTAAATCAT | AGCTTTTAGAGGCAGTGAATCAATGGGCCATTCTTT | ATCCTTTCGTTCTTCTCCGCGTCCAGCTAAACCATT | TATTTTTTCAGGTTTGGCGGGTTTATCGACTGCAAA | TTGCTTGAAGCAAGAGATGTTTTAGGTGGAAAGGTT |
| #29 | green | TGAGTGCATTGAACTTGAGCTGGCATAGTAAATCAT | AGCTTTTAGAGGCAGTGAATCAATGGGCCATTCTTT | ATCCTTTCGTTCTTCTCCGCGTCCAGCTAAACCATT | TATTTTTTCAGGTTTGGCGGGTTTATCGACTGCAAA | TTGCTTGAAGCAAGAGATGTTTTAGGTGGAAAGGTT |
| | | TGAGTGCATTGAACTTGAGCTGGCATAGTAAATCAT | AGCTTTTAGAGGCAGTGAATCAATGGGCCATTCTTT | ATCCTTTCGTTCTTCTCCGCGTCCAGCTAAACCATT | TATTTTTTCAGGTTTGGCGGGTTTATCGACTGCAAA | TTGCTTGAAGCAAGAGATGTTTTAGGTGGAAAGGTT |
| #30 | green | TGAGTGCATTGAACTTGAGCTGGCATAGTAAATCAT | AGCTTTTAGAGGCAGTGAATCAATGGGCCATTCTTT | ATCCTTTCGTTCTTCTCCGCGTCCAGCTAAACCATT | TATTTTTTCAGGTTTGGCGGGTTTATCGACTGCAAA | TTGCTTGAAGCAAGAGATGTTTTAGGTGGAAAGGTT |
| | | TGAGTGCATTGAACTTGAGCTGGCATAGTAAATCAT | AGCTTTTAGAGGCAGTGAATCAATGGGCCATTCTTT | ATCCTTTCGTTCTTCTCCGCGTCCAGCTAAACCATT | TATTTTTTCAGGTTTGGCGGGTTTATCGACTGCAAA | TTGCTTGAAGCAAGAGATGTTTTAGGTGGAAAGGTT |
